# An economic and disease transmission model of human papillomavirus and oropharyngeal cancer in Texas

**DOI:** 10.1038/s41598-021-81375-5

**Published:** 2021-01-19

**Authors:** Chengxue Zhong, Li Xu, Ho-Lan Peng, Samantha Tam, Li Xu, Kristina R. Dahlstrom, Chi-Fang Wu, Shuangshuang Fu, Wenyaw Chan, Erich M. Sturgis, Lois M. Ramondetta, Libin Rong, David R. Lairson, Hongyu Miao

**Affiliations:** 1 Department of Biostatistics and Data Science, School of Public Health, The University of Texas Health Science Center at Houston, 1200 Pressler Street, Houston, TX 77030 USA; 2 Department of Statistics, School of Mathematical and Statistics, Guangdong University of Foreign Studies, Xiaoguwei Street, Guangzhou, Guangdong China; 3 Department of Management, Policy, and Community Health, School of Public Health, The University of Texas Health Science Center at Houston, 1200 Pressler Street, Houston, TX USA; 4 Department of Otolaryngology, Henry Ford Health System, 2799 W Grand Blvd, Detroit, MI USA; 5 Department of Head and Neck Surgery, Division of Surgery, The University of Texas MD Anderson Cancer Center, 1515 Holcombe Blvd, Houston, TX USA; 6 Department of Epidemiology, Human Genetics and Environmental Sciences, School of Public Health, The University of Texas Health Science Center at Houston, 1200 Pressler Street, Houston, TX USA; 7 Division of Cancer Prevention and Population Sciences, Department of Epidemiology, The University of Texas MD Anderson Cancer Center, 1400 Pressler St, Houston, TX USA; 8 Department of Gynecologic Oncology and Reproductive Medicine, The University of Texas MD Anderson Cancer Center, 1515 Holcombe Blvd., Houston, TX USA; 9 Department of Mathematics, University of Florida, 1400 Stadium Rd, Gainesville, FL USA

**Keywords:** Applied mathematics, Health care economics, Epidemiology

## Abstract

In 2017, 46,157 and 3,127 new oropharyngeal cancer (OPC) cases were reported in the U.S. and Texas, respectively. About 70% of OPC were attributed to human papillomavirus (HPV). However, only 51% of U.S. and 43.5% of Texas adolescents have completed the HPV vaccine series. Therefore, modeling the demographic dynamics and transmission of HPV and OPC progression is needed for accurate estimation of the economic and epidemiological impacts of HPV vaccine in a geographic area. An age-structured population dynamic model was developed for the U.S. state of Texas. With Texas-specific model parameters calibrated, this model described the dynamics of HPV-associated OPC in Texas. Parameters for the Year 2010 were used as the initial values, and the prediction for Year 2012 was compared with the real age-specific incidence rates in 23 age groups for model validation. The validated model was applied to predict 100-year age-adjusted incidence rates. The public health benefits of HPV vaccine uptake were evaluated by computer simulation. Compared with current vaccination program, increasing vaccine uptake rates by 50% would decrease the cumulative cases by 4403, within 100 years. The incremental cost-effectiveness ratio of this strategy was $94,518 per quality-adjusted life year (QALY) gained. Increasing the vaccine uptake rate by 50% can: (i) reduce the incidence rates of OPC among both males and females; (ii) improve the quality-adjusted life years for both males and females; (iii) be cost-effective and has the potential to provide tremendous public health benefits in Texas.

## Introduction

Human papillomavirus (HPV) is etiologically linked with several cancers, including oropharyngeal cancer (OPC). In 2017, 46,157 and 3,127 new OPC cases were reported in the United States and Texas^1^. In the United States, more than 70% of OPC cases are attributable to HPV^2^. Over the past decades, the incidence of HPV-related OPC has been increasing and is now higher than that of HPV-related cervical cancer^3^. HPV-related OPC incidence differs by sex in the United States, with overall incidence rates for men being two to four times higher than those for women^4^. Since there is currently no validated screening tool for OPC, primary prevention is the most effective available public health strategy for reducing the burden of HPV-related OPC in the United States.

Prophylactic vaccination against HPV is associated with a significant decrease in the prevalence of oral infection by HPV types that can cause OPC among young adults^5^, and thus has the potential to protect against most of the HPV-related OPCs. The HPV vaccine has been shown to be long-lasting; multiple studies have shown sustained effectiveness and high immunogenicity for at least 10 years after vaccination^6–8^. Although HPV-vaccine trials were not designed to evaluate oropharyngeal end-points, data from the Costa Rica vaccine trial showed 93% (95% CI 63 to 100%) protection against prevalent oral HPV infection, similar to the efficacy for cervical lesions^9–11^. The Advisory Committee on Immunization Practices of the U.S. Centers for Disease Control and Prevention (CDC) currently recommends routine vaccination of boys and girls aged 11 to 12 years against HPV; vaccination may be started as young as age 9 years^12^. Catch-up vaccination for all individuals up to age 26 years is also recommended. Vaccination of adults aged 26 years and older is considered of limited public health benefit; however, vaccination of adults aged 27–45 years who are not adequately vaccinated may benefit and is recommended through shared clinical decision-making^12^. HPV vaccination rates remain low in the United States, with approximately half (54%) of adolescents having completed the vaccine series in 2018 from the most recent estimate available^13^. Meanwhile, only 48% of Texas teenagers received a complete series of HPV vaccine, which is much lower than the vaccine uptake rate in the National Immunization Survey-Teen (NIS-Teen) 2019, Texas^14^. To determine the societal benefit of dedicating resources to public health policies, including those related to immunization, policy makers and stakeholders rely on cost-effectiveness and disease burden studies. Such studies rely on accurate and up-to-date mathematical models.

In general, modeling HPV-related cancers is a challenging task for a number of reasons, including, for example, the need for a sophisticated model structure, with numerous model variables and unknown parameters, and poor data availability. Particularly for HPV-related OPC, there is little to no previous modeling work on OPC progression. To our knowledge, this is the first attempt to develop and apply a comprehensive mathematical model to quantitatively understand HPV-related OPC incidence and its economic consequence in Texas. For this purpose, an age-structured population model of the natural history of HPV-related OPC was developed for this study, based on the literature and our current understanding of demographic dynamics, disease progression, and incidence rate of OPC. Whenever possible, Texas data were utilized to model the disease, to model the behavior of at-risk individuals, and to validate the model. The mathematical model incorporated HPV infection, progression, treatment, and other characteristics; strategies for vaccination; and the epidemiologic and economic effects of HPV vaccination. Also, given the heterogeneities among different age groups, age structures were explicitly incorporated into the ordinary differential equation models^15, 16^.

The primary aim of this paper was to develop and apply a model for comparing the OPC-related health and economic consequences of proposed investments for increasing the HPV vaccination rates in Texas over 100 years. While there are well-developed HPV vaccination economic models for the nation that have been utilized to inform the recommendations of the CDC^17^, large diverse states such as Texas require information relevant to their populations in order to inform state-level decision-makers about the health and economic consequences of public health disease-prevention investments. This paper provides valuable information for Texas and an example for other states to consider.

## Results

### Model validation

The model consisted of a large system of ordinary differential equations (ODEs) similar to the previous work of Elbasha and Peng^18, 19^, which provided a solid foundation for depicting the population dynamics, HPV transmission, and vaccination simultaneously. In this study, the epidemiologic model of HPV-associated OPC was incorporated and the predicted HPV-associated OPC incidence rates were used for model validation.

**Figure 1:**
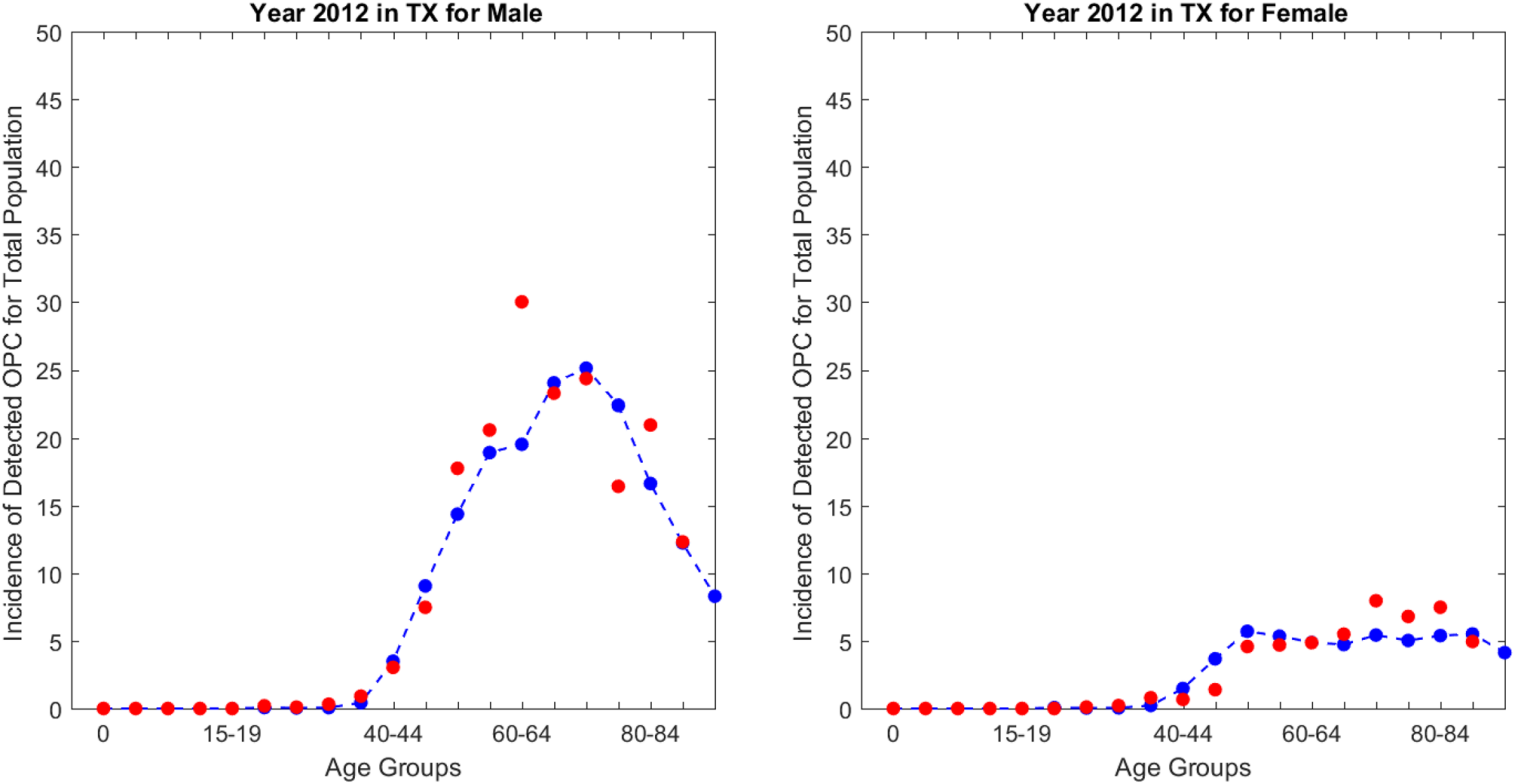
Model validation using real 2012 oropharyngeal cancer (OPC) incidence data for males and females in Texas (TX). Red points denoted real values and blue points denoted predicted values.

The parameters and initial variables collected from Year 2010 in Texas used to validate the model. The predicted age-specific incidence rates (per 100,000) compared with the actual data for Years 2011 and 2012 in Texas, and both the real incidence rates and the predicted incidence rates were calculated (Fig. 1). The two curves matched each other reasonably well and had similar trends for all age groups, although the match was better for 2012 than for 2011, especially for males older than 45 years (Fig. S4). For males, the predicted result was slightly larger than the actual data for the Texas population older than 55 years; for females, the difference observed only for the population older than 60 years.

### Prediction

**Figure 2:**
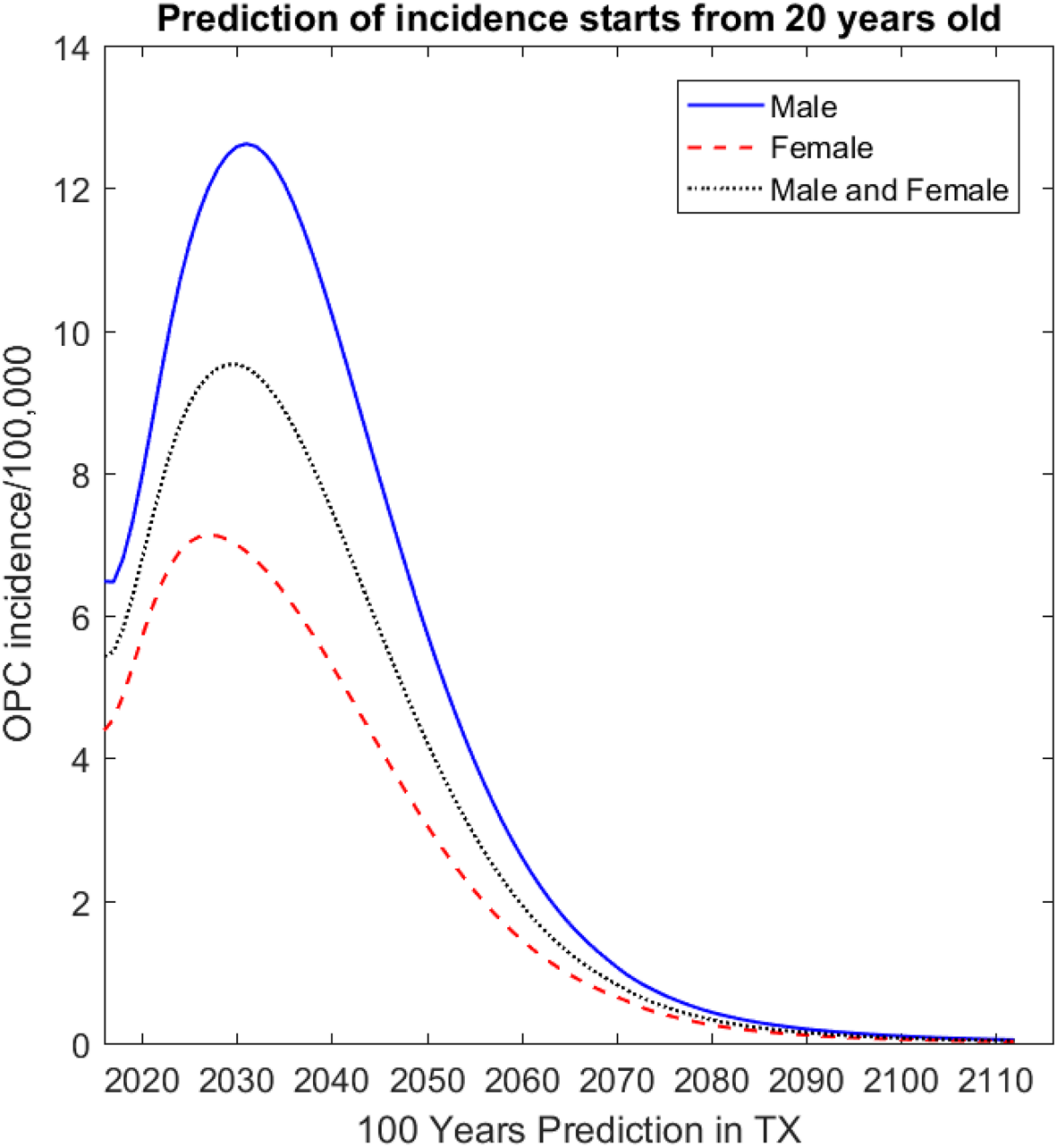
Prediction by the model of oropharyngeal cancer (OPC) incidence for Texas (TX) for 100 years, beginning at age 20 years.

Age-adjusted incidence rates predicted for 100 years and all age groups were included. While other modelers such as Chesson et al.^20^ set the starting age group at 12 years, Graham et al.^21^ listed the vaccine target age for starting HPV vaccination for females in the U.S. at 11 years. Therefore, age-adjusted incidence rate was predicted by covering age groups started from 11 years (Supplemental Fig. S5). Because disease transmission occurs mainly among adults, our age-adjusted incidence rate prediction figure starts at age 20 years (Fig. 2).

The initial parameter values were determined for 2015 and calibrated for prediction of age-adjusted incidence rates. The parameter values fixed for the 100-year study time horizon. The predicted incidence rate (per 100,000) increased until Year 2030 and then declined for the remainder of the time span. The predicted male incidence rates were much higher than for females, which clearly reflects the known differences in OPC between genders. The predicted incidence rates covered Years 2019 to Year 2115 (the results for years 2016 to 2018 found to be very sensitive to the initial variable values and thus may be artifacts).

Cumulative cases prevented in Texas are presented in Table 1. Although increasing the vaccination coverage rate by 50% for females only did not affect males directly, vaccinating females resulted in a notable decrease in OPC among males. For example, increasing the vaccine uptake rate of females decreased the respective cumulative number of OPC cases for males and females by 1271 and 1297, respectively, within 100 years. If vaccination uptake rates increased for both females and males, providing both direct and indirect protection, even more cases of disease among men were prevented; for example, 2362 cases among men and 1450 cases among women were prevented after 100 years of vaccination. Additionally, more cases expected prevented with a longer follow-up time. For instance, compared with 10 years, the number of OPC cases prevented among men and women was around 25 times and 20 times higher, respectively, over 100 years when vaccination coverage rate was increased for males and females.

**Table 1:** Cumulative cases prevented by different strategies.

	Males				Females			
	10 years	20 years	50 years	100 years	10 years	20 years	50 years	100 years
**Strategy 1: vaccine uptake rate 50% increase for females only**								
OPC	88	341	1108	1271	132	464	1129	1297
**Strategy 2: vaccine uptake rate 50% increase for females and males**								
OPC	183	804	3126	3632	202	815	2379	2747

Female and male vaccination includes age 13 to 26 years.

### Policy assessment and sensitivity analysis of model parameters

Policy focuses on the health gains from investments to increase the vaccination uptake rate among the target groups in Texas. We assessed the impact of different vaccination strategies or scenarios on health and economic consequences, such as HPV-related OPC incidence rates, years of life, QALYs, and costs. Incremental cost-effectiveness ratios (ICERs) estimated for different vaccination coverage rates, vaccination covered age groups, and whether male vaccination rates increased in addition to those of females. The effects of rate of sexual partner change and detection rate of the OPC parameters on health and economic outcomes are unknown or highly uncertain and therefore assessed with sensitivity analysis.

#### Vaccination coverage rate

Vaccination adherence is one of the most important factors in determining the level of disease prevention. In our model, age and gender specific vaccination uptake rates, including uptake rates of first dose and second dose, were collected from CDC^22^ and the values were presented in Supplements Text S2 and S3. In addition to predicting the impact of vaccination with first dose of vaccine, we considered the adherence to the second vaccine dose. Results derived for increasing the first and second vaccine uptake rates by multiplying 1.5 to each value of vaccine uptake rate respectively or simultaneously. As the model predicted results since 2016, the intervention started in Year 2016.

##### First dose

**Figure 3:**
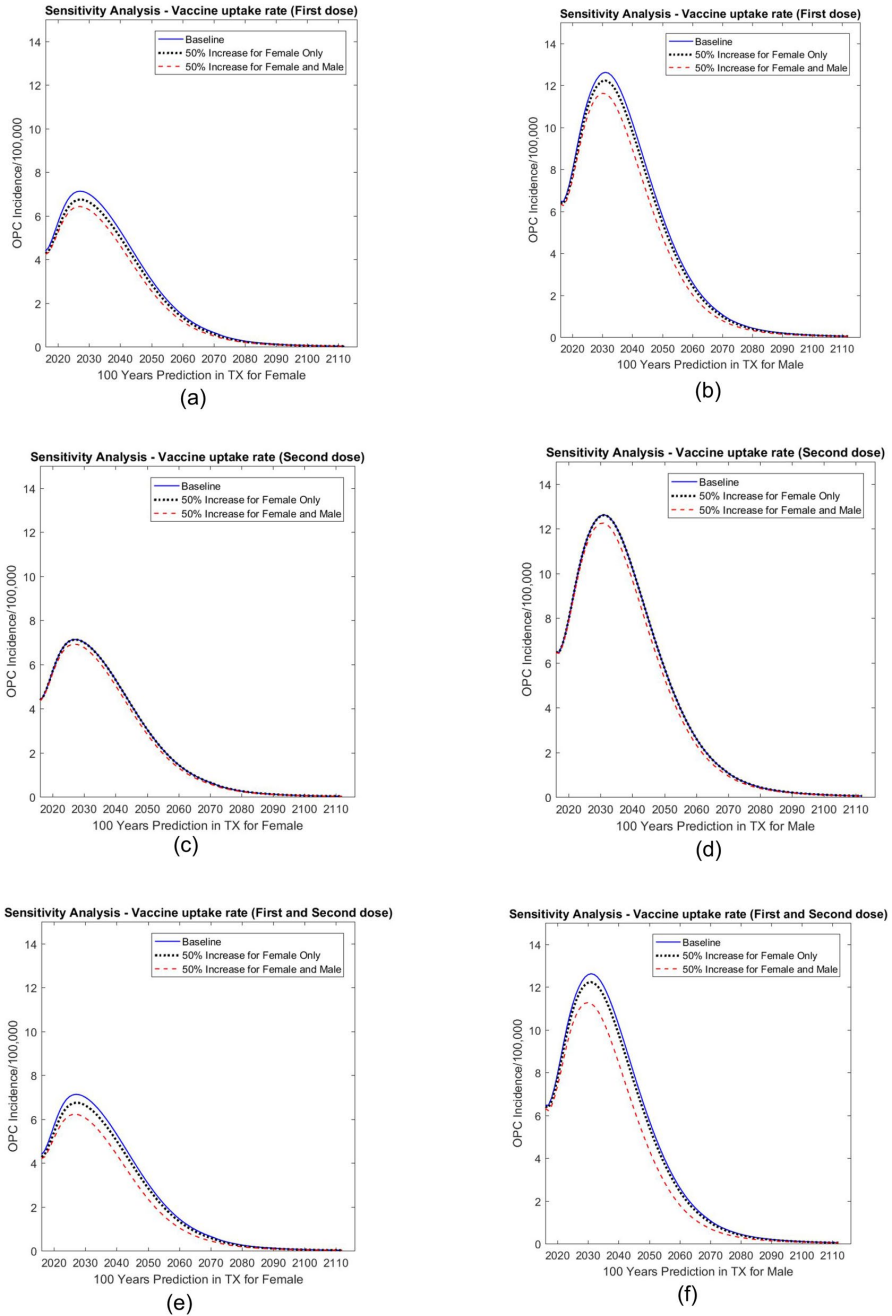
Sensitivity analysis: predicted effect of first dose (**a**, **b**), second dose (**c**, **d**), and first plus second dose (**e**, **f**) vaccine coverage rates on oropharyngeal cancer (OPC) incidence in Texas (TX). The graph on the left shows predictions for females; the graph on the right shows predictions for males.

As shown in Fig. 3a and b, when the first dose vaccine uptake rate increased 50% for females only, the incidence rate (per 100,000) decreased by 2.79% for males and 5.30% for females by Year 2031 compared with the baseline predictions. When the first dose vaccine uptake increased by 50% for both males and females, the incidence rate (per 100,000) decreased by 6.91% for males and 10.04% for females by Year 2031, again compared with the baseline predictions.

##### Second dose

By changing the proportion of those with uptake receiving two doses, the incidence rate changed less than the incidence rate changes when we varied the first dose uptake rate (Fig. 3c,d). Compared with the baseline results, the incidence rate decreased by 0.10% for males and 0.12% for females by Year 2031 when we increased the vaccination uptake rate of females only; the incidence rates decreased by 2.36% for males and 3.15% for females at Year 2031 if uptake rates of both females and males were increased.

##### First and second dose

We also performed sensitivity analysis by changing the vaccine uptake rate for the first and second doses at the same time. As shown in Fig. 3e and f, the incidence rate (per 100,000) decreased by 2.89% for males and 5.44% for females by Year 2031 when we increased the vaccine coverage for females only. The incidence rate (per 100,000) decreased by 9.31% for males and 13.05% for females by Year 2031 when we increased the vaccine coverage rate 50% for both males and females.

#### Vaccination starting age

**Figure 4:**
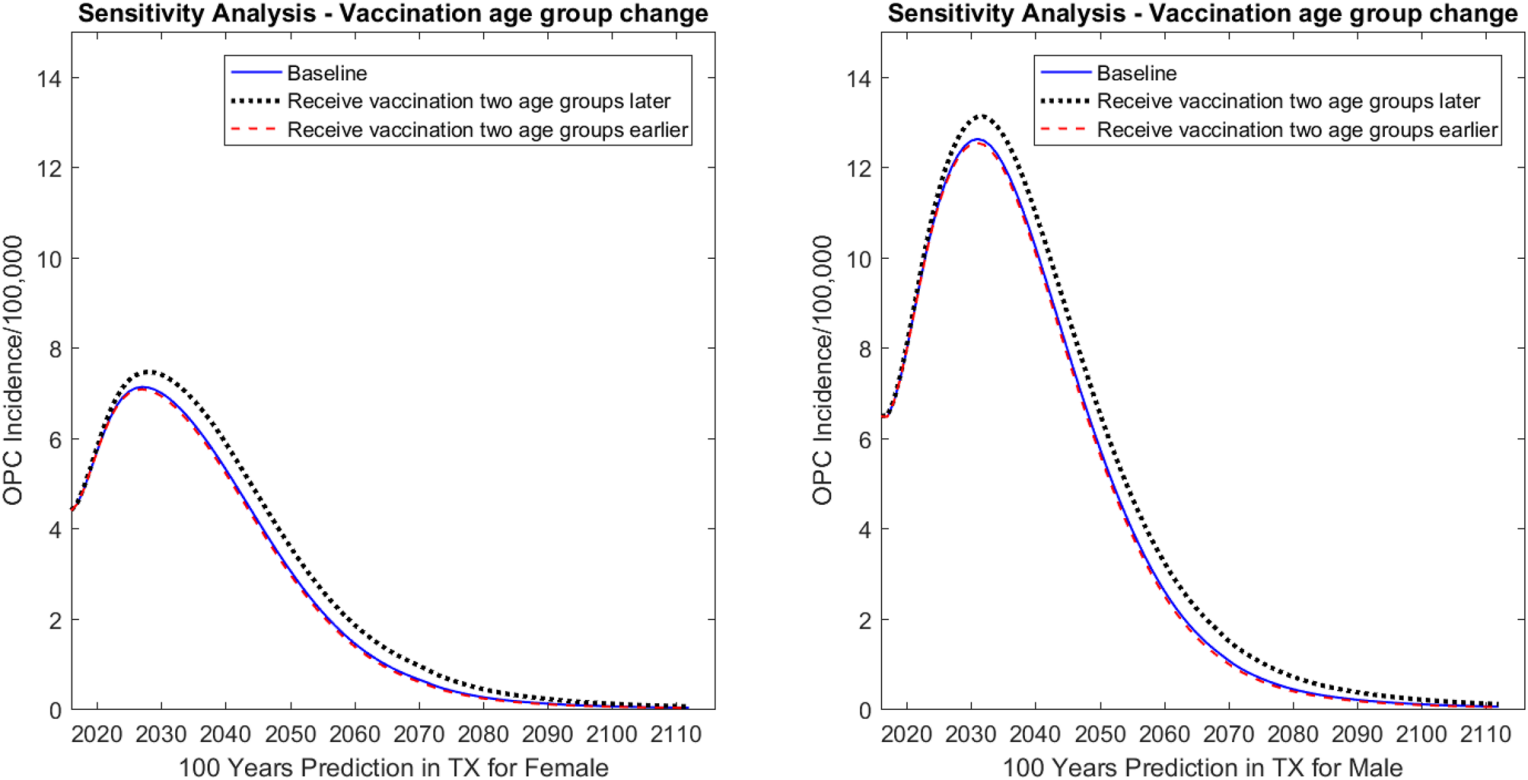
Sensitivity analysis: predicted effect of vaccination covered age groups on oropharyngeal cancer (OPC) incidence in Texas (TX). The graph on the left shows predictions for females; the graph on the right shows predictions for males.

The starting age of vaccine uptake has a notable effect on incidence rate. We predicted the incidence rates for three different scenarios: (1) the current vaccination coverage strategy, starting from age 13–14 years for both males and females, (2) assuming that people would receive vaccination two age groups earlier, that is, age 9–10 years for males and females, and (3) assuming that people would receive vaccination two age groups later, that is, age 18 years for males and females (Fig. 4). Compared with the baseline results, the incidence rates (per 100,000) increased by 3.11% for males and 4.98% for females by Year 2031 when the vaccination started two age groups later. When the vaccination started two age groups earlier, the predicted incidence rates (per 100,000) were almost the same as the baseline results for both males and females over 100 years, with a decrease of 0.54% and 0.85%, respectively.

#### Rate of sexual partner change

**Figure 5:**
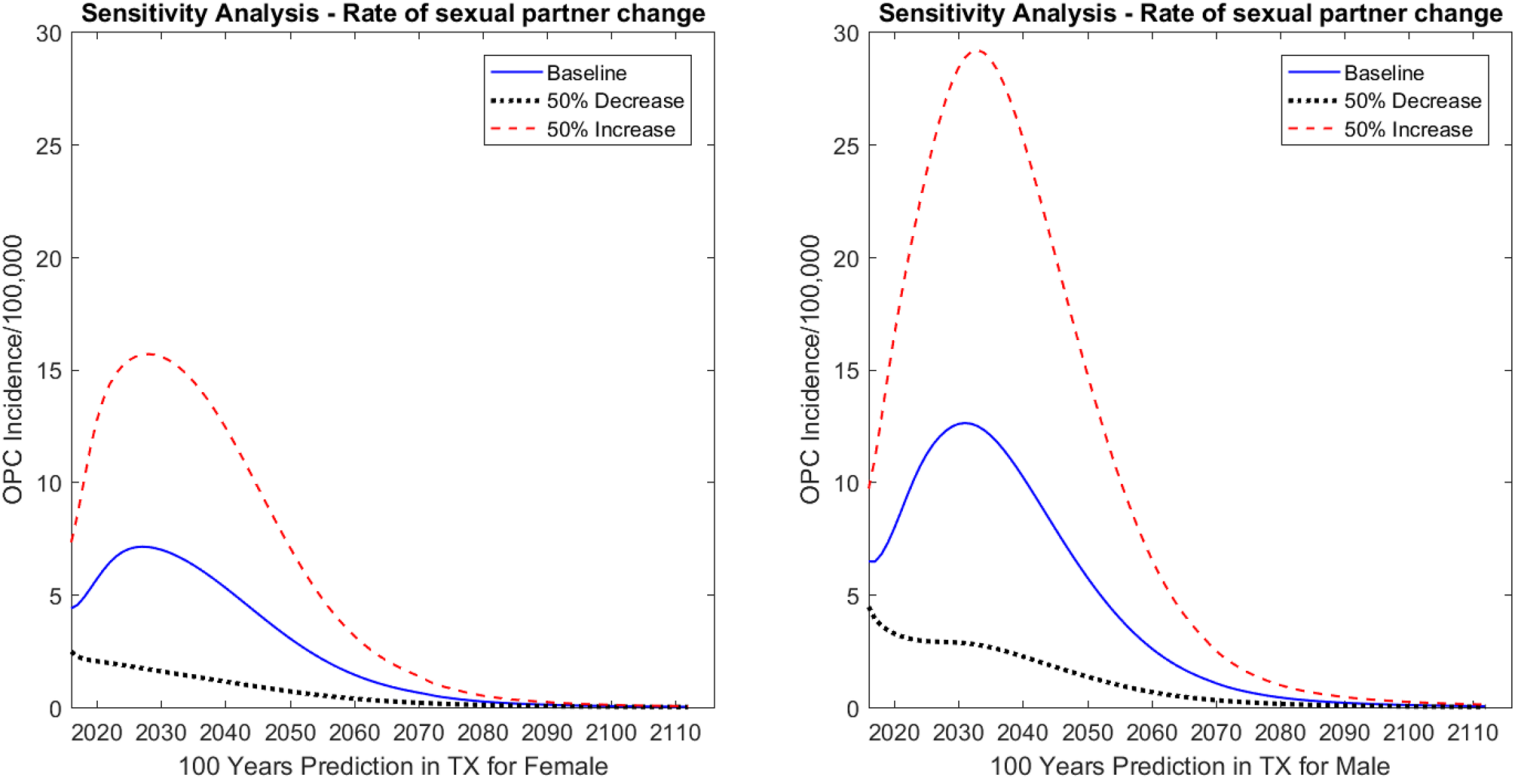
Sensitivity analysis: predicted effect of rate of sexual partner change on oropharyngeal cancer (OPC) incidence in Texas (TX). The graph on the left shows predictions for females; the graph on the right shows predictions for males.

Sexual behavior is an important contributor to HPV-related OPC incidence rate^18^. We increased and decreased the rate of sexual partner change by 50% from baseline; Fig. 5 shows that the rate of sexual partner change had the expected effects on the incidence rates. When the rate of sexual partner change decreased by 50%, in Year 2031 the incidence dropped by 4.53 cases (per 100,000) for males and 2.48 cases (per 100,000) for females compared with the baseline results. When the rate of sexual partner change increased by 50%, the incidence increased by 7.12 cases (per 100,000) for males and 3.93 cases (per 100,000) for females in Texas at Year 2031 compared with the baseline results.

#### Detection rate of OPC

**Figure 6:**
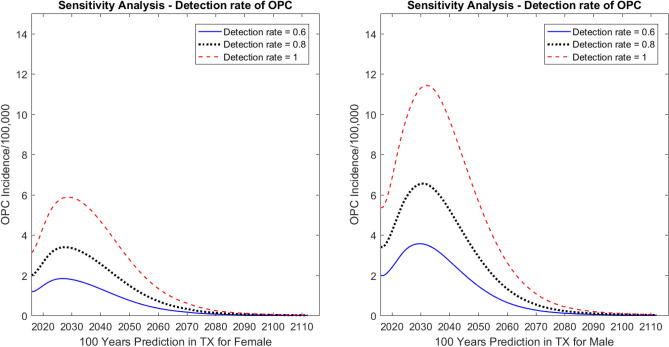
Sensitivity analysis: predicted effect of detection rate on oropharyngeal cancer (OPC) incidence in Texas (TX). The graph on the left shows predictions for females; the graph on the right shows predictions for males.

Because of the unavailability of detection rates for OPC, we performed sensitivity analyses to assess the impact of detection rates on the disease incidence predictions. Figure 6 shows that when the detection rate was set at 0.8, the incidence rates increased by 2.32 cases (per 100,000) for males and 1.30 cases (per 100,000) for females in Year 2031 compared with the detection rate of 0.6. When the detection rate was set at 1, the incidence rates increased by 5.94 cases (per 100,000) for males and 3.36 cases (per 100,000) for females in Year 2031 compared with the detection rate of 0.6.

### Economics

#### Incremental cost-effectiveness ratio

Table 1 shows the cumulative cases of OPC that prevented by HPV vaccine in the Texas population over time. The effects of increasing vaccine uptake rate were remarkable over time, with larger impact on males. Compared to the current vaccine policy, increasing the rate for female only saved 88 and 132 OPC cases in a 10-year time span for male and female, respectively. Within a 100-year time span, the prevented OPC cases were 1271 and 1297 for male and female, respectively. Increasing vaccine uptake rate for both females and males saved 183 and 202 OPC cases in 10 years for male and female, respectively, and 3632 and 2747 cases in 100 years.

Table 2 shows the short-term to long-term vaccine costs, promotion costs, and treatment costs. There was no tremendous increase for vaccine costs (0.99% in 10 years and 0.77% in 100 years) if uptake rate was increased 50% for females only. However, the extra costs were notable if the uptake rate increased 50% for both males and females, with a 6.89% increase in 10 years and a 4.33% increase in 100 years. The promotion costs were the major part of vaccination cost when we increased vaccine uptake rate: $1.6 billion in 10 years for increasing female only rate and $3.3 billion for increasing both gender rates. However, with the increase of vaccine uptake rate, the treatment costs would decline by 2.27% for the female only increase strategy and 3.87% for the both gender increase strategy in 10 years; the treatment cost would decrease by 3.94% and 9.20% in 100 years.

**Table 2:** Short-term to long-term costs under different scenarios.

Time span	Cost	Baseline	50% Increase females	50% Increase males and females	Percentage change: strategy 1 vs. baseline (%)	Percentage change: strategy 2 vs. baseline (%)
10 years	Vaccine cost	$1,215,970,837	$1,228,036,081	$1,299,696,369	0.99	6.89
	Promotion cost	$0	$1,610,203,300	$3,288,240,276		
	Treatment cost*	$790,679,741	$772,723,638	$760,056,563	− 2.27	− 3.87
20 years	Vaccine cost	$1,979,363,530	$1,996,366,114	$2,086,066,593	0.86	5.39
	Promotion cost	$0	$3,012,153,182	$6,110,436,523		
	Treatment cost*	$1,752,402,352	$1,697,131,568	$1,643,925,198	− 3.15	− 6.19
50 years	Vaccine cost	$3,244,954,345	$3,270,506,029	$3,392,724,426	0.79	4.55
	Promotion cost	$0	$5,445,831,584	$10,903,328,729		
	Treatment cost*	$2,890,132,667	$2,779,265,735	$2,633,107,535	− 3.84	− 8.89
100 years	Vaccine cost	$3,916,784,811	$3,946,784,282	$4,086,414,163	0.77	4.33
	Promotion cost	$0	$6,751,642,838	$13,455,242,628		
	Treatment cost*	$2,949,072,752	$2,832,929,791	$2,677,776,292	− 3.94	− 9.20

(1) Discount rate is 0.03; (2) Costs reported in 2018 USD; (3) Female and male vaccination includes age 13 to 26 years.

Table 3 provides results for the cost-effectiveness analysis, which compared three vaccination coverage levels and three vaccination costs. The criteria for a strategy being cost-effective followed the convention of $100,000 per QALY gained^23^. For each vaccination scenario, the strategies ordered from least resource intensive (i.e., current vaccine uptake rate) in the top row to the most resource intensive (i.e., increase vaccination uptake rate 50% for males and females) in the bottom row. We varied combinations of vaccine acquisition cost and vaccine promotion uptake costs in a sensitivity analysis of the ICERs. Base-case vaccination cost values were derived from CDC data ($178.14 for the vaccine; $50.64 and $20.26 for promoting uptake of the first and second dose, respectively)^24^. The best-case cost assumptions ($142.51 for vaccine; $20.26 for extra first dose and second dose costs) and the worst-case cost assumptions ($217.11 per dose for vaccine; $87.92 and $20.26 for promoting first dose and second dose) were calculated^25^. The base-case results showed that changing from a strategy of a 50% increase over current vaccination levels among females only to a strategy of a 50% increase among females and males (Age-specific base vaccine uptake rates were presented in presented in Supplements Text S2 and S3) reduced the ICER from $126,689.20/QALY to $94,517.75/QALY. The variation of vaccine costs also had an important impact on the ICERs. For example, the ICERs increased to $194,065.98/QALY (baseline vs. strategy 1) and $144,497.70/QALY (strategy 1 vs. strategy 2) when the vaccination cost was $217.11 and dropped to $71,734.45/QALY and $53,718.93/QALY when the vaccination cost was $142.51.

**Table 3:** Incremental cost per quality-adjusted life year (QALY) gained by vaccination for vaccine uptake rate 50% increase scenario.

Coverage strategy	Total cost*	Lost-QALYs	Incremental cost	Lost-QALYs difference	$/QALY
**Base case vaccine cost: $178.14; intervention cost: $50.64 for the first dose; $20.26 for the second dose**					
Baseline	$6,865,857,562	1,281,115			
50% increase over current rate for females	$13,531,356,911	1,228,503	$6,665,499,349	52,612	$126,689.20
50% increase over current rate for males and females	$20,219,433,083	1,157,743	$6,688,076,172	70,760	$94,517.75
**Best case vaccine cost: $142.512; intervention cost: $20.26 for the first dose; $20.26 for the second dose**					
Baseline	$6,082,500,600	1,281,115			
50% increase females	$9,856,665,333	1,228,503	$3,774,164,732	52,612	$71,734.45
50% increase males and females	$13,657,816,653	1,157,743	$3,801,151,321	70,760	$53,718.93
**Worst case vaccine cost: $217.11; intervention cost: $87.92 for the first dose; $20.26 for the second dose**					
Baseline	$7,722,695,466	1,281,115			
50% increase females	$17,933,089,043	1,228,503	$10,210,393,577	52,612	$194,065.98
50% increase males and females	$28,157,746,183	1,157,743	$10,224,657,141	70,760	$144,497.70

(1) Discount rate is 0.03; (2) Costs reported in 2018 USD; (3) Planning horizon is 100 years; (4) Female and male vaccination includes age 13 to 26 years.

*Note that "Total cost" denotes the sum of vaccination cost, promotion cost and treatment cost.

To assess the impact of uncertainty of some model parameters on the ICERs, other scenarios were considered, such as no discounting of costs and QALYs or no promotion costs for the expansion of vaccine uptake (Table 4). In almost all scenarios, a strategy of increasing vaccine uptake by 50% for females only was dominated by the strategy of increasing both female and male vaccine uptake by 50%, except in the setting with no promotion cost and vaccination cost of $217.11. The strategy of adding male vaccination uptake rate was cost-effective in most of scenarios, especially for the setting of no discounting and no promotion cost. Finally, we also computed long-term ICER results for Years of Life Saved and the short-term to long-term costs, QALYs, and ICERs (See Supplemental Tables S3, S4). Table S4 showed that the increase of vaccination uptake rate became cost-effective after 50 years with the threshold of $100,000 per QALY and strategy of adding female and male dominated strategy of increasing female only after 50 years.

**Table 4:** Sensitivity analysis: estimated cost per quality-adjusted life years ($ per QALY) gained when other model parameters are varied.

Parameter varied in sensitivity analysis in addition to varying vaccine uptake rate	50% increase females	50% increase males and females
Base case (vaccine cost = $178.14)	$126,689.20	$94,517.75
Lower vaccination cost per dose (vaccine cost = $142.51)	$71,734.45	$53,718.93
Higher vaccination cost per dose (vaccine cost = $217.11)	$194,065.98	$144,497.70
Assuming no discount (vaccine cost = $178.14)	$182,489.66	$124,332.14
Assuming no discount (vaccine cost = $142.51)	$103,726.46	$70,802.06
Assuming no discount (vaccine cost = $217.11)	$279,060.27	$189,923.46
Assuming no promotion cost (vaccine cost = $178.14)	− $1637.32	− $219.39
Assuming no promotion cost (vaccine cost = $142.51)	− $1751.36	− $614.05
Assuming no promotion cost (vaccine cost = $217.11)	− $1512.58	$212.29

(1) Discount rate is 0.03; (2) Costs reported in 2018 USD; (3) Planning horizon is 100 years; (4) Female and male vaccination includes age 13 to 26 years.

#### Cost-effectiveness acceptability curve

The uncertainty in the results of the cost-effectiveness analysis were summarized in CEACs, which are provided in Supplemental Figs. S6 and S7. The curves indicated the percent of simulations for which the strategy was cost-effective for a determined monetary value that the policy-maker would be willing to pay for a QALY saved. For instance, the CEAC for changing the vaccination coverage rate showed that increasing uptake rate for females only was cost-effective in all simulations if the willingness to pay was higher than $135,000 per QALY. And all simulations were cost-effective when the uptake rate was extended to include males at this monetary threshold. With willingness to pay reached $100,000 per QALY, the strategy of increasing female and male vaccine uptake rates was cost-effective in all the simulations.

Relative to the commonly reported threshold of $100,000 to $150,000 per QALY saved^23^, the results suggest that substantially increasing vaccination against HPV was very cost-effective, especially when the vaccination uptake rate was increased among males and females. The efficiency of increasing vaccination of males was driven by their relatively high and increasing OPC incidence rates.

## Discussion

In this study, we built an age-structured HPV infection model to estimate the impact of HPV vaccination on OPC progression in Texas. We obtained model parameter values from the literature or calibrated them with data from Year 2010 and Year 2015 in Texas. The age-specific incidence rates predicted by the model validated against OPC incidence for Years 2011 and 2012 in Texas, and they matched the real data well. The model then applied to predict the age-adjusted incidence rate for Texas until 2115. Multiple sensitivity analyses conducted on several of the parameters, including vaccine uptake rate, coverage age and sexual activity parameters, to determine the impact of HPV vaccination and sexual behavior on disease progression.

Our work quantified the trend of OPC incidence rates for Texas. The model showed an expected incremental increase on predicted incidence rate for the first 20 years and then a decline for the rest of prediction period (80 years). While estimated tends in OPC incidence rates and the cost-effectiveness of increasing vaccination rates for boys and girls were similar to previous estimates of U.S. trends, there were some differences in the Texas results. A Canadian study^21^ examined the cost-effectiveness of immunization of 12-year-old boys with a Markov model that did not account for herd immunity. With vaccine efficacy at 99 percent and 70 percent uptake, 0.05 QALYs and $145 estimated saved per person in the target population, or $28 mil over the lifetime of the cohort of 192,940 individuals. Vaccination of boys continued to be cost saving when efficacy and uptake were assumed to be 50 percent, but total savings declined to $8 mil. in Canada.

This study has some limitations. First, the study focused on only one of several diseases related to HPV infection. We focused on OPC because its incidence is increasing and it has overtaken cervical cancer as the leading health problem likely to decrease due to HPV immunization. Researchers have demonstrated the value of preventing other cancers, genital warts, and recurrent respiratory papillomatosis with HPV vaccination^26, 27^. Addition of these diagnoses to the current work would make the increased investment in vaccination even more cost-effective and possibly cost saving, as shown in the work by investigators at the CDC^17^. Second, we did not attempt model fitting because of poor data availability and the lack of efficient regression techniques for large-scale ordinary differential equation models. Therefore, the parameter values obtained from the literature or were calibrated based on the actual data and assumptions. A better match would be possible if model fitting becomes feasible in the future. Third, the vaccination uptake rate held constant during the 100-year prediction period, which may not reflect the real evolution of vaccine policy and vaccination behavior. Fourth, because of the lack of population data, the validation was limited to only data for 2011 and 2012 in Texas. With more data in the future, we will improve model validation. For example, the predicted age-specific incidence rate is a little higher than the actual incidence rate in people aged 60 to 79 years, and the difference becomes smaller in people aged 80 years and older. Fifth, there is no dependable literature on the rates of detection of OPC. In one study^28^, the detection rate for oral cancer was 86%. We estimated that the detection rates for local and regional stages of OPC were lower than 86% and assumed the detection rate for distant stage OPC as 1. Therefore, detection rates were assumed 0.68, 0.85, and 1 for local, regional, and distant OPC. Finally, the predicted incidence rate declined briefly in the beginning of the prediction period; this drop may have resulted from the initial conditions.

## Conclusion

The paper adds to the literature on health/economic policy analysis of HPV immunization by adapting a comprehensive population disease model for a state-level assessment of the long-term health and economic consequences of increasing adherence to HPV immunization guidelines. We incorporated up-to-date treatment and vaccine cost estimates for OPC based on Texas health insurance claims, published reports, up-to-date disease and sexual behavior data and cost estimates for promoting vaccine uptake among males and females to achieve a 50% increase in the HPV vaccination rates. Thus, this work provides information that may be actionable at the state level of government to decide resource allocation regarding the prevention of HPV-related conditions compared to other state investment opportunities in health. Finally, this work may provide a template for similar studies in other states that contemplate HPV-related public health investments.

## Method

### Age-structured model

Previous modeling work by Elbasha et al.^18, 29^ provided a solid foundation for a comprehensive mathematical model to depict population growth, HPV transmission, and vaccination. This modeling framework included a demographic model, epidemiological model, and the natural history of OPC for different sex and age groups. Model parameter notations, definitions, and the source of parameters presented in Table 5; and Table 6 contains initial variables’ notations, definitions, and the sources of initial values. Our complete model equations provided in Supplementary Text S1; additionally, model structure diagrams given in Supplementary Figs. S1–S3. While the basic structure of our model follows the published model^18, 29^, the natural history of OPC was modeled and incorporated, and the entire framework was updated and adapted to the Texas population for making specific predictions for Texas. In the demographic model, people transfer from their current age group to the next age group, except for the oldest age group (≥ 85 years), at age- and gender-specific rates. For instance, the calibrated transfer rate for Texas suggested that about 12.4% of females from the second age group (age 1–8 years) move to the third age group (age 9–10 years) each year, compared to 13.4% of males. The population size for every age group depended on the probability of transferring to the next age group and the probability of transferring from the younger group, and on the OPC-related and non-OPC-related death rates. The population growth for the first age group (0–1 years) calculated based on the birth rate because there is no younger age group, and there was no transfer out for the oldest age group.

**Table 5:** Summary of variables.

Variable	Unit	Reference	Description
X	Persons		Susceptible
V1	Persons	^44, 45^	Vaccinated with 1 dose
V2	Persons	^44, 45^	Vaccinated with 2 doses
VS	Persons		Vaccinated with waned immunity
Y	Persons		Infected persons
U	Persons		Persistently infected
ZS	Persons		Recovered without seroconversion
Z	Persons	^42, 43^	Recovered with seroconversion
WS	Persons		Infected, vaccinated with waned immunity
W1	Persons	^41^	Infected, vaccinated with 1 dose
W2	Persons	^41^	Infected, vaccinated with 2 doses
PS	Persons	^41^	Persistently infected, vaccinated
P1	Persons	^41^	Persistently infected, vaccinated with 1 dose
P2	Persons	^41^	Persistently infected, vaccinated with 2 doses
QS	Persons		Recovered, vaccinated without seroconversion
Q	Persons	^42, 43^	Recovered, vaccinated with seroconversion
Hx	Persons		Population with tonsillectomy
Hy	Persons		Population with tonsillectomy that are infected
Hz	Persons	^42, 43^	Population with tonsillectomy that were infected, recovered, seroconverted
Hzs	Persons		Population with tonsillectomy that were infected, recovered, not seroconverted
Hv1	Persons	^44, 45^	Vaccinated with 1 dose, with tonsillectomy
Hv2	Persons	^44, 45^	Vaccinated with 2 doses, with tonsillectomy
Hvs	Persons		Waned immunity, with tonsillectomy
Hw	Persons	^41^	Infected, vaccinated, with tonsillectomy
Hqs	Persons		Recovered, vaccinated without seroconversion, with tonsillectomy
Hq	Persons	^42, 43^	Recovered, vaccinated with seroconversion, with tonsillectomy
N	Persons		Total number of persons
DOPCl	Cases/100,000	Detected local oropharyngeal cancer	
DOPCr	Cases/100,000	Detected regional oropharyngeal cancer	
DOPCd	Cases/100,000	Detected distant oropharyngeal cancer	
SOPC	Proportion		Oropharyngeal cancer survivors

**Table 6:** Initial variables: definitions and sources of initial values (HPV, human papillomavirus; OPC, oropharyngeal cancer; DOPC, detected OPC; L, local; R, regional; D, distant; USD, U.S. dollars).

Parameters	Unit	Reference	Description
B	Persons		New borne
Δ	Per 100 population	^46^	Rate of tonsillectomy
λ	Case per year		Force of infection
sc	Partners per year		Rate of sexual partner change
$\epsilon$_1_ $\epsilon$_2_ $\epsilon$_3_	%		Degree of assortative mixing between age and sexual activity groups
pc_l_, pa_i_	Partners per year		Relative partner acquisition rate
$\stackrel{-}{{c}_{j}}$	Partners per year		Mean partner acquisition rate
χ	Proportion	^47^	Rate of local oropharyngeal cancer-associated death (L:1, R:2, D:3)
d	Change in group per year	^48^	Transfer rate
q	Annual population change		Annual growth rate
µ	Cases per person	^49^	Death rate
pL	% detection		Detection rate of local oropharyngeal cancer
pR	% detection		Detection rate of regional oropharyngeal cancer
pD	% detection		Detection rate of distant oropharyngeal cancer
σz	%		Rate of waning immunity following recovery with seroconversion
σzs	%		Rate of waning immunity following recovery without seroconversion
ϒ	%	^50^	Rate of recovery from HPV infection
prf	%	^50^	Proportion of infections that are destined to be persistent
θsz	%	^39, 41^	Reactivation rate following sero-conversion
θszs	%	^30–32^	Reactivation rate, who did not sero-convert
ι	%	^42, 43^	Probability of sero-conversion following HPV clearance
ψz	%		Degree of protection following sero-conversion
ψzs	%		Degree of protection following no sero-conversion
ψp_1_	% protection		Degree of protection following sero-conversion, vaccinated with 1 dose
ψp_2_	% protection		Degree of protection following sero-conversion, vaccinated with 2 dose
φ	%		Proportion of new born vaccinated
φc	persons	^44, 45^	Vaccine uptake rate with first dose
Φ1	%		Proportion receiving only 1 dose
Φ2	%		Proportion receiving only 2 dose
ψv_1_	%	^51^	Degree of protection with 1 dose
ψv_2_	%		Degree of protection with 2 doses
σv_1_	%		Rate of waning immunity following 1 dose vaccination
σv_2_	%		Rate of waning immunity following 2 dose vaccination
σq	%		Rate of waning immunity following recovery with seroconversion
σqs	%		Rate of waning immunity following recovery without seroconversion
α	%	^52^	Relative rate of recovery from breakthrough infection
θt_L_	Per year		Rate of progression from HPV infection to local OPC
θt_R_	Per year		Rate of progression from local OPC to regional OPC
θt_D_	Per year		Rate of progression from regional OPC to distance OPC
θp_1_	Per year		Rate of progression from breakthrough infection to DOPC (with 1 dose)
θp_2_	Per year		Rate of progression from breakthrough infection to DOPC (with 2 doses)
θp_s_	Per year		Rate of progression to DOPC in patients that are persistently infected and vaccinated
θh_y_	Per year		Rate of progression from HPV infection (persistent) to DOPC in patients with tonsillectomy that are infected
θh_w_	Per year		Rate of progression from HPV infection (persistent) to DOPC in patients with tonsillectomy that are infected vaccinated
Ω	Cases per year		Cure rate of local oropharyngeal cancer(L:1, R:2, D:3)
θtw_1_	Per year		Rate of progression to DOPC in patients that are vaccinated with 1 dose, then are infected
θtw_2_	Per year		Rate of progression to DOPC in patients that are vaccinated with 2 doses, then are infected
θtw_s_	Per year		Rate of progression to DOPC in patients that are infected, vaccinated and have waning immunity
ψq	% protection		Degree of protection following recovery of an infection in previously vaccinated individuals with seroconversion
ψqs	% protection		Degree of protection following recovery of an infection in previously vaccinated individuals without seroconversion
θsq	%		Reactivation rate in patients who are recovered, vaccinated and seroconverted
θsqs	%		Reactivation rate in patients who are recovered, vaccinated and no seroconversion
ξ	%	^19, 24, 31^	Discount rate
T	Year		Planning horizon quality %
quality	%		Quality of life weights for a normal individual
qopc	%		Quality of life weights for an individual in OPC
θ_prev_	Per patient over lifetime (USD)		Cost of treating for all detected opc patients(prevalence)
θ_inc_	Per patient in first year of diagnosis (USD)		Cost of treating for new detected opc patients(incidence)
vaccine	Cost of each dose of vaccine (USD)		Cost of the vaccine

In the epidemiological model, HPV transmission specified by gender, age, and sexual activity. The important subpopulations related to HPV transmission included the susceptible population, infected population, persistently infected population, vaccinated population, infectious vaccinated population, persistently infected vaccinated population, and recovered vaccinated population. Additionally, since the three-dose HPV vaccination strategy has been changed into a two-dose strategy recently, the force of HPV infection (λ) was redefined by^19^, which was determined by the number of sexual partners, relative infectivity of vaccine breakthrough cases, and probability of people being in different groups.

In the OPC natural history model, the OPC stage contained detected local, regional, and distant cases and survived cases. We updated parameters of published models to reflect current information on HPV transmission and OPC progression. For OPC progression, equations now reflect the epidemiologic variations among different stages of detected OPC. The population of individuals with detected local OPC determined by progression rate, detection rate, and various subpopulations, as given below:

$$\begin{aligned} DOPCl_{l,1,c}^{{\prime}} \left[ t \right] = \, & p_{L} *\theta_{tL} *Y_{l,1,c} \left( t \right) + p_{L} * \left( {\theta_{p1} *P1_{l,1,c} \left( t \right) + \theta_{p2} *P2_{l,1,c} \left( t \right) + \theta_{ps} *PS_{l,1,c} \left( t \right)} \right) \\ & + p_{L} * \left( {\theta_{tw1} *W1_{l,1,c} \left( t \right) + \theta_{tw2} *W2_{l,1,c} \left( t \right) + \theta_{tws} *WS_{l,1,c} \left( t \right)} \right) \\ & + p_{L} * \theta * U_{l,1,c} + p_{L} * \theta_{hw} * Hw_{l,1,c} + p_{L} * \theta_{hy} * Hy_{l,1,c} \\ & - \left( {\mu_{1,c} + d_{1,c} + \chi_{1,1} + \Omega_{L} + \theta_{tR} } \right)* DOPCl_{l,1,c} \left[ t \right] \\ \end{aligned}$$

$$\begin{aligned} DOPCl_{l,i,c}^{{\prime}} \left[ t \right] = \, & d_{i - 1,c} *DOPCl_{l,i - 1,c} \left[ t \right] + p_{L} *\theta_{tL} *Y_{l,i,c} \left( t \right) \\ & + p_{L} * \left( {\theta_{p1} *P1_{l,i,c} \left( t \right) + \theta_{p2} *P2_{l,i,c} \left( t \right) + \theta_{ps} *PS_{l,i,c} \left( t \right)} \right) \\ & \; + p_{L} * \left( {\theta_{tw1} *W1_{l,i,c} \left( t \right) + \theta_{tw2} *W2_{l,i,c} \left( t \right) + \theta_{tws} *WS_{l,i,c} \left( t \right)} \right) \\ & + p_{L} * \theta * U_{l,i,c} + p_{L} * \theta_{hw} * Hw_{l,i,c} + p_{L} * \theta_{hy} * Hy_{l,i,c} \\ & \; - \left( {\mu_{i,c} + d_{i,c} + \chi_{1,i} + {\Omega }_{L} + \theta_{tR} } \right)* DOPCl_{l,i,c} \left[ t \right] \\ \end{aligned}$$

where $l$ denotes sexual activity group, $c$ denotes gender, and $i$ denotes age group. Parameter ${p}_{L}$ denotes the detection rate of local OPC and ${p}_{R}$ and ${p}_{D}$ denote the detection the rates of regional and distant cancers, respectively. We multiplied all subpopulations by their detection rates to predict the prevalence of detected OPC. Here, $d$ was the population transfer rate and $i=1, 2, 3, \dots , 23$. As no younger group exists when $i=1$ (age group 0–1 years), there is a small difference between the two equations. When $i$ was greater than 1, the individuals with detected local OPC were from the detected local OPC cases in the first age group, HPV-infected individuals, persistently infected vaccinated individuals, infected vaccinated individuals with waned immunity, persistently infected individuals, infected vaccinated individuals who had undergone tonsillectomy, and infected vaccinated individuals who had not undergone tonsillectomy. We subtracted the population that died of any cause except OPC, the population that transferred to the next age group, the population that died of OPC, the population cured of OPC, and the population with detected local OPC that developed regional OPC; θ_tR_ represented the progression rate from local to regional status.

Equations given below model how the disease develops from local to regional:

$$\begin{aligned} DOPCr_{l,1,c}^{{\prime}} \left[ t \right] = \, & p_{R} \times \theta_{tL} \times Y_{l,1,c} \left( t \right) + p_{R} \times \theta_{tR} \times DOPCl_{l,1,c} \\ & + p_{R} \times \left( {\theta_{p1} \times P1_{l,1,c} \left( t \right) + \theta_{p2} \times P2_{l,1,c} \left( t \right) + \theta_{ps} \times PS_{l,1,c} \left( t \right)} \right) \\ & + p_{R} \times \left( {\theta_{tw1} \times W1_{l,1,c} \left( t \right) + \theta_{tw2} \times W2_{l,1,c} \left( t \right) + \theta_{tws} \times WS_{l,1,c} \left( t \right)} \right) \\ & + p_{R} \times \theta \times U_{l,1,c} + p_{R} \times \theta_{hw} \times Hw_{l,1,c} + p_{R} \times \theta_{hy} \times Hy_{l,1,c} \\ & - \left( {\mu_{1,c} + d_{1,c} + \chi_{2,1} + {\Omega }_{R} + \theta_{tD} } \right) \times DOPCr_{l,1,c} \left[ t \right] \\ \end{aligned}$$

$$\begin{aligned} DOPCr_{l,i,c}^{{\prime}} \left[ t \right] = \, & d_{i - 1,c} \times DOPCr_{l,i - 1,c} \left[ t \right] + p_{R} \times \theta_{tR} \times DOPCl_{l,i,c} + p_{L} \times \theta_{tL} \times Y_{l,i,c} \left( {\text{t}} \right) \\ & + p_{R} \times \left( {\theta_{p1} \times P1_{l,i,c} \left( t \right) + \theta_{p2} \times P2_{l,i,c} \left( t \right) + \theta_{ps} \times PS_{l,i,c} \left( t \right)} \right) \\ & + p_{R} \times \left( {\theta_{tw1} \times W1_{l,i,c} \left( t \right) + \theta_{tw2} \times W2_{l,i,c} \left( t \right) + \theta_{tws} \times WS_{l,i,c} \left( t \right)} \right) \\ & + p_{R} \times \theta \times U_{l,i,c} + p_{R} \times \theta_{hw} \times Hw_{l,i,c} + p_{R} \times \theta_{hy} \times Hy_{l,i,c} \\ & - \left( {\mu_{i,c} + d_{i,c} + \chi_{2,i} + {\Omega }_{R} + \theta_{tD} } \right) \times DOPCr_{l,i,c} \left[ t \right] \\ \end{aligned}$$

For the detected regional disease, when the disease progressed from regional to distant, we used the following equations:

$$\begin{aligned} DOPCd_{l,1,c}^{{\prime}} \left[ t \right] = \, & p_{D} \times \theta_{tL} \times Y_{l,1,c} \left( t \right) + p_{D} \times \theta_{tD} \times DOPCr_{l,1,c} \\ & + p_{D} \times \left( {\theta_{p1} \times P1_{l,1,c} \left( t \right) + \theta_{p2} \times P2_{l,1,c} \left( t \right) + \theta_{ps} \times PS_{l,1,c} \left( t \right)} \right) \\ & + p_{D} \times \left( {\theta_{tw1} \times W1_{l,1,c} \left( t \right) + \theta_{tw2} \times W2_{l,1,c} \left( t \right) + \theta_{tws} \times WS_{l,1,c} \left( t \right)} \right) \\ & + p_{D} \times \theta \times U_{l,1,c} + p_{D} \times \theta_{hw} \times Hw_{l,1,c} + p_{D} \times \theta_{hy} \times Hy_{l,1,c} \\ & - \left( {\mu_{1,c} + d_{1,c} + \chi_{3,1} + {\Omega }_{D} } \right) \times DOPCd_{l,1,c} \left[ t \right] \\ \end{aligned}$$

$$\begin{aligned} DOPCd_{l,i,c}^{{\prime}} \left[ t \right] = \, & d_{i - 1,c} \times DOPCd_{l,i - 1,c} \left[ t \right] + p_{D} \times \theta_{tD} \times DOPCr_{l,i,c} + p_{D} \times \theta_{tL} \times Y_{l,i,c} \left( {\text{t}} \right) \\ & + p_{D} \times \left( {\theta_{p1} \times P1_{l,i,c} \left( t \right) + \theta_{p2} \times P2_{l,i,c} \left( t \right) + \theta_{ps} \times PS_{l,i,c} \left( t \right)} \right) \\ & + p_{D} \times \left( {\theta_{tw1} \times W1_{l,i,c} \left( t \right) + \theta_{tw2} \times W2_{l,i,c} \left( t \right) + \theta_{tws} \times WS_{l,i,c} \left( t \right)} \right) \\ & + p_{D} \times \theta \times U_{l,i,c} + p_{D} \times \theta_{hw} \times Hw_{l,i,c} + p_{D} \times \theta_{hy} \times Hy_{l,i,c} \\ & - \left( {\mu_{i,c} + d_{i,c} + \chi_{3,i} + {\Omega }_{D} } \right)* DOPCd_{l,i,c} \left[ t \right] \\ \end{aligned}$$

In the equations, θ_tL_, θ_tR_, and θ_tD_ represent the progression rates for three stages for an individual with OPC. Specifically, θ_tL_ denotes the progression rate from HPV infection to local OPC, θ_tR_ denotes the progression rate from local to regional OPC, and θ_tD_ denotes the progression rate from regional to distant OPC. The median time of disease progression in all age groups was around 8 months^30^. The progression rates were age-specific, as the disease usually progresses more quickly in the older groups, and the θ_tR_ and θ_tD_ were larger than the θ_tL_ since the disease progression accelerates when the disease progresses to regional or distant status compared with local status.

### Economics

Applying the 2010 and 2015 Texas base vaccination rates^31–33^, the incremental cost-effectiveness ratios (ICERs) were calculated for 50% increases in the female vaccination rate only and in the female and male rates (Age-specific base vaccine uptake rates presented in Supplements Text S2 and S3). The 50% increase was an important and feasible target and was also examined in a Canadian analysis of HPV immunization expansion^21^. Fifty percent increase in immunization would move Texas close to the 2020 Health People goal for the U.S.^34^. ICERs were computed by dividing the net economic costs (vaccine plus vaccination promotion costs minus cancer treatment cost averted) by life years and quality-adjusted life years (QALY) gained. Sensitivity analyses altering the vaccination strategies and HPV transmission rates examined the impact of behavior and vaccination on the incidence of OPC. To estimate the ICERs for the vaccination strategies, we calculated the vaccination cost, treatment cost, and QALYs based on the following formulas. All costs valued in 2018 U.S. dollars.

#### Vaccination cost

$$\begin{aligned} {\text{Vaccinate}}_{{\text{a}}} \left( {\text{t}} \right) = \, & {\text{ vaccine}}\;\; \times \sum \limits_{{\text{l}}} \sum \limits_{{\text{i}}} \sum \limits_{{\text{c}}} \left[ {B_{l,c} \times \phi_{l,c} + {\Phi }_{1} \times \phi_{l,i,c} \times \left( {Y_{l,i,c} + X_{l,i,c} + U_{l,i,c} } \right)} \right] \\ & + \;{\text{vaccine}} \times \sum \limits_{{\text{l}}} \sum \limits_{{\text{i}}} \sum \limits_{{\text{c}}} \left[ {{\Phi }_{2} \times \phi_{l,i,c} \times \left( {Y_{l,i,c} + X_{l,i,c} + U_{l,i,c} } \right)} \right] \\ & + \;cost_{pro\_1} \times pop_{1} + cost_{pro\_2} \times pop\_2 \\ \end{aligned}$$

Total vaccination costs included the unit cost per vaccination and the number of people vaccinated. Let $${B}_{l,c}$$ denote the newborn population; φ_l,c_ denote the proportion of newborns vaccinated; φ_l,i,c_ represent the vaccine uptake rate for the first dose; Φ_1_ and Φ_2_ represent the proportion of receiving only one dose or two dose vaccine respectively; and Y_l,i,c_, X_l,i,c_, U_l,i,c,_ denote the population vaccinated^18^. The cost of the HPV vaccine was $178.14 per dose^25^. $$cos{t}_{pro\_1}$$ and $$cos{t}_{pro\_2}$$ represent the extra costs for motivating people to obtain the immunization and complete the second dose, and they were included in the vaccination cost calculation; the estimated costs for promoting the first and second doses were $50.64 and $20.26, respectively^35–37^. $$po{p}_{1}$$ and $$po{p}_{2}$$ denote the corresponding target population for promotion. Sensitivity analysis assessed the ICER estimates over a range of vaccination costs and efficacies.

#### Treatment cost

$$Trea{t}_{a}\left(t\right)= cos{t}_{opc}\times incidence$$

The per-case cost of OPC treatment was $151,622^38^. Cost estimated from experience of a retrospective cohort of 467 Texas patients with OPC. The Truven MarketScan database used to identify commercially insured patients with OPC newly diagnosed during the period 2011 to 2014. All dollars adjusted to year 2015 values using the Consumer Price Index from the U.S. Bureau of Labor Statistics. A generalized linear model estimated the total healthcare cost for OPC patients.

#### Effectiveness: quality-adjusted life years

The lost quality-adjusted life years due to OPC calculated using the following equation:

$${\mathrm{QALY}}_{\mathrm{a}}={\int }_{0}^{T}[{{\sum }_{l}{\sum }_{i}{\sum }_{c}{q}_{c,i}\times (\left(1-qop{c}_{l}\right)\times SOP{C}_{l,i,c}+\left(1-qop{c}_{l}\right)\times DOPC{l}_{l,i,c}+\left(1-qop{c}_{r}\right)\times DOPC{r}_{l,i,c}+\left(1-qop{c}_{d}\right)\times DOPC{d}_{l,i,c})]\times e}^{-\xi t}$$

where $qop{c}_{l}, qop{c}_{r} and qop{c}_{d}$ denote the quality of life weights for subjects in the health status being categorized as OPC local, regional, and distant, respectively. The value of quality of life weights listed in Supplements Text S2 and S3 for Year 2010 and 2015 respectively. $\xi$ represents the discount rate; all costs and QALYs were discounted to current value at an annual discount rate of 3%^29^. T denotes the time span, a 100-year analytic horizon.

#### Cost-effectiveness ratio

To compare vaccination strategies $\mathrm{a}$ and $\mathrm{a{^{\prime}}}$, the ICER was calculated as follows:

$$\frac{{\mathrm{Cost}}_{\mathrm{a}}-{\mathrm{Cost}}_{{\mathrm{a}}^{{^{\prime}}}}}{{\mathrm{QALY}}_{\mathrm{lost},\mathrm{a}}-{\mathrm{QALY}}_{\mathrm{lost},{\mathrm{a}}^{{{\prime}}}}}$$

Here ${\mathrm{Cost}}_{\mathrm{a}}={\int }_{0}^{\mathrm{T}}(Vaccinat{e}_{a}\left(t\right)+Trea{t}_{a}\left(t\right))$, and the corresponding value for vaccination strategy a’ was ${\mathrm{Cost}}_{{a}^{^{\prime}}}={\int }_{0}^{\mathrm{T}}(Vaccinat{e}_{{a}^{^{\prime}}}\left(t\right)+Trea{t}_{{a}^{^{\prime}}}\left(t\right))$. Commonly, the lost quality-adjusted life years of the baseline strategy was larger than the strategy of increasing female vaccination or increasing vaccination for both genders, and the cost of the baseline strategy was smaller than the other improvement strategies. In practice, ${\mathrm{QALY}}_{\mathrm{lost},,{\mathrm{a}}^{{^{\prime}}}}-{\mathrm{QALY}}_{\mathrm{lost},\mathrm{a}}$ applied instead of ${\mathrm{QALY}}_{\mathrm{lost},\mathrm{a}}-{\mathrm{QALY}}_{\mathrm{lost},{\mathrm{a}}^{{^{\prime}}}}$.

#### Probabilistic sensitivity analysis

We computed the cost-effectiveness acceptability curve (CEAC) by performing probabilistic sensitivity analysis. Parameters for vaccine uptake rate and quality of life for patients with OPC were included in probabilistic sensitivity analyses. For quality of life, beta distribution was applied to generate 1000 random samples as inputs (α = 68.3, β = 27.24). For vaccine uptake rate, the Latin Hypercube sampling technique based on multivariate normal distribution conducted to generate random samples as input values. Because of the computational complexity, only 100 random samples for each parameter were generated as inputs in the simulation.

### Model initial variables, parameters, and calibration

All of the values of variables, parameters were listed in Supplementary Text S2 (Year 2010) and S3 (Year 2015). For parameters that were not available in the literature, we calibrated from reasonable data or knowledge of HPV infection and the epidemiology of OPC. Seroconversion following oral HPV infection appears to be rare among both males and females^39–43^. The HPV vaccines function primarily by inducing durable systemic virus-neutralizing antibody responses, thereby preventing infection and possibly guarding against reinfection and disease^13, 21^. Therefore, we assumed the corresponding parameters (ls, θ_tw1_, θ_tw2_, θ_tws_, θ_tp1_, θ_tp2_, θ_tps_) to be 0.

### Implementation and computation

The model structure was implemented in MATLAB (MathWorks, Natick, MA) and the ode15s solver was applied to obtain numeric solutions. Both the relative and the absolute error tolerances were set as 1.0 × 10^–7^ in the ordinary differential equation solver; we also set maximum step size as 1.0 × 10^–2^ and restricted the total number of persons (variable N) as non-negative.

Simulations were performed for the Texas population. The entire population was stratified into 23 age groups, two gender groups, and three sexual activity groups. The variable values of initial conditions were from Year 2010 in Texas and we calculated the age-specific incidence rates (per 100,000) and age-adjusted incidence rates (per 100,000) for validation, prediction and sensitivity analyses.

To check the validity of the model, we compared the predicted age-specific incidence rates (per 100,000) with the real OPC incidence rates (per 100,000) from the Surveillance, Epidemiology, and End Results Program (SEER) database for Texas and the Texas Cancer Registry database. Because of limitations on data availability, comparisons conducted for Texas in 2011 and 2012 only. One-way sensitivity analyses implemented by changing the value of some parameters according to pre-specified ranges.

## Supplementary Information

Supplementary Legends.

Supplementary Text S1.

Supplementary Text S2.

Supplementary Text S3.

Supplementary Figure S1.

Supplementary Figure S2.

Supplementary Figure S3.

Supplementary Figure S4.

Supplementary Figure S5.

Supplementary Figure S6.

Supplementary Figure S7.

Supplementary Table S1.

Supplementary Table S2.

Supplementary Table S3.

Supplementary Table S4.

## Data Availability

All the data used in this study are from public sources.
