# Supplementary material for: An economic and disease transmission model of human papillomavirus and oropharyngeal cancer in Texas
Source: Sci Rep. 2021 Jan 19;11:1802. doi: 10.1038/s41598-021-81375-5 (PMC7815750; doi:10.1038/s41598-021-81375-5)
Supplement: Supplementary file 1 — Supplementary Legends. [file 41598_2021_81375_MOESM1_ESM.pdf]

Supplemental for: An Economic and Disease Transmission Model of Human Papillomavirus and Oropharyngeal Cancer in Texas

Chengxue Zhong, Li Xu, Ho-Lan Peng, Samantha Tam, Li Xu, Kristina R. Dahlstrom, Chi-Fang Wu, Shuangshuang Fu, Wenyaw Chan, Erich M. Sturgis, Lois M. Ramondetta, Libin Rong, David R. Lairson, Hongyu Miao

The following supplementary materials are provided:

- Text S1 (PDF)
- Text S2 (PDF)
- Text S3 (PDF)
- Figure S1 (PDF)
- Figure S2 (PDF)
- Figure S3 (PDF)
- Figure S4 (PDF)
- Figure S5 (PDF)
- Figure S6 (PDF)
- Figure S7 (PDF)
- Table S1 (Excel)
- Table S2 (Excel)
- Table S3 (Excel)
- Table S4 (Excel)
