# Supplementary material for: An economic and disease transmission model of human papillomavirus and oropharyngeal cancer in Texas
Source: Sci Rep. 2021 Jan 19;11:1802. doi: 10.1038/s41598-021-81375-5 (PMC7815750; doi:10.1038/s41598-021-81375-5)
Supplement: Supplementary file 2 — Supplementary Text S1. [file 41598_2021_81375_MOESM2_ESM.pdf]

Supplemental for: Modeling HPV-related Oropharyngeal Cancer in Texas with Cost Effectiveness Prediction

Text S1: ODE Models for OPC

Susceptible persons : X

$$X_{l,1,c}{}'[t]$$
$$= B_{l,c} * (1 - \phi_{l,c}) + \sigma z_{1,c} * Z_{l,1,c}[t] + \sigma zs_{1,c} * ZS_{l,1,c}[t] - (\lambda_{l,1,c} + \phi c_{l,1,c} + d_{1,c} + \mu_{1,c} + \Delta_1) * X_{l,1,c}[t]$$

$$X_{l,i,c}{}'[t]$$
$$= d_{i-1,c} * X_{l,i-1,c}[t] + \sigma z_{i,c} * Z_{l,i,c}[t] + \sigma zs_{i,c} * ZS_{l,i,c}[t] - (\lambda_{l,i,c} + \phi c_{l,i,c} + d_{i,c} + \mu_{i,c} + \Delta_i) * X_{l,i,c}[t]$$

Vaccinated persons with one dose: V1

$$V1_{l,1,c}{}'[t]$$
$$= B_{l,c} * \Phi_{1,c} * \phi_{l,c} + \Phi_{1,c} * \phi c_{l,1,c} * (X_{l,1,c}[t] + Z_{l,1,c}[t] + ZS_{l,1,c}[t])$$
$$-((1 - \text{prf}) * (1 - \psi v_c^I) * \lambda_{l,1,c} + \text{prf} * (1 - \psi p_{1,c}) * \lambda_{l,1,c} + \mu_{1,c} + d_{1,c} + \sigma v_{1,c}^I + \Delta_i) * V1_{l,1,c}[t]$$

$$V1_{l,i,c}{}'[t]$$
$$= d_{i-1,c} * V1_{l,i-1,c}[t] + \Phi_{1,c} * \phi c_{l,i,c} * (X_{l,i,c}[t] + Z_{l,i,c}[t] + ZS_{l,i,c}[t])$$
$$-((1 - \text{prf}) * (1 - \psi v_c^I) * \lambda_{l,i,c} + \text{prf} * (1 - \psi p_{1,c}) * \lambda_{l,i,c} + \mu_{i,c} + d_{i,c} + \sigma v_{i,c}^I + \Delta_i) * V1_{l,i,c}[t]$$

Vaccinated persons with two dose: V2

$$V2_{l,1,c}{}'[t]$$
$$= B_{l,c} * \Phi_{2,c} * \phi_{l,c} + \Phi_{2,c} * \phi c_{l,1,c} * (X_{l,1,c}[t] + Z_{l,1,c}[t] + ZS_{l,1,c}[t])$$
$$-((1 - \text{prf}) * (1 - \psi v_c^{II}) * \lambda_{l,1,c} + \text{prf} * (1 - \psi p_{2,c} * \lambda_{l,1,c} + \mu_{1,c} + d_{1,c} + \sigma v_{1,c}^{II} + \Delta_i) * V2_{l,1,c}[t]$$

$$V2_{l,i,c}{}'[t]$$
$$= d_{i-1,c} * V2_{l,i-1}[t] + \Phi_{2,c} * \phi c_{l,i,c} * (X_{l,i,c}[t] + Z_{l,i,c}[t] + ZS_{l,i,c}[t])$$
$$-((1 - \text{prf}) * (1 - \psi v_c^{II}) * \lambda_{l,i,c} + \text{prf} * (1 - \psi p_{2,c}) * \lambda_{l,i,c} + \mu_{i,c} + d_{i,c} + \sigma v_{i,c}^{II} + \Delta_i) * V2_{l,i,c}[t]$$

Vaccinated persons with waned immunity: VS

$$VS_{l,1,c}{}'[t]$$
$$= \sigma v_{1,c}^I * V1_{l,1,c}[t] + \sigma v_{1,c}^{II} * V2_{l,1,c}[t] + \sigma q_{1,c} * Q_{l,1,c}[t]$$
$$+\sigma qs_{1,c} * QS_{l,1,c}[t] - (\lambda_{l,1,c} + d_{1,c} + \mu_{1,c} + \Delta_1) * VS_{l,1,c}[t]$$

$$VS_{l,i,c}{}'[t]$$
$$= d_{i-1,c} * VS_{l,i-1,c}[t] + \sigma v_{i,c}^I * V1_{l,i,c}[t] + \sigma v_{i,c}^{II} * V2_{l,i,c}[t] + \sigma q_{i,c} * Q_{l,i,c}[t]$$

$+\sigma qs_{i,c} * QS_{l,i,c}[t] - (\lambda_{l,i,c} + d_{i,c} + \mu_{i,c} + \Delta_i) * VS_{l,i,c}[t]$

Infected persons : Y

$Y_{l,1,c}{}'[t]$
$= (1 - \text{prf}) * \lambda_{l,1,c} * X_{l,1,c}[t] + ((1 - \psi z_{1,c}) * \lambda_{l,1,c} + \theta s z_{1,c}) * Z_{l,1,c}[t]$
$+((1 - \psi zs_{1,c}) * \lambda_{l,1,c} + \theta szs_{1,c}) * ZS_{l,1,c}[t] - (\mu_{1,c} + d_{1,c} + \gamma_{1,c} + \theta_{tL} + \Delta_1) * Y_{l,1,c}[t]$

$Y_{l,i,c}{}'[t]$
$= d_{i-1,c} * Y_{l,i-1,c}[t] + (1 - \text{prf}) * \lambda_{l,i,c} * X_{l,i,c}[t] + ((1 - \psi z_{i,c}) * \lambda_{l,i,c} + \theta s z_{i,c}) * Z_{l,i,c}[t]$
$+((1 - \psi zs_{i,c}) * \lambda_{l,i,c} + \theta szs_{i,c}) * ZS_{l,i,c}[t] - (\mu_{i,c} + d_{i,c} + \gamma_{i,c} + \theta_{tL} + \Delta_i) * Y_{l,i,c}[t]$

Persistently Infected persons : U

$i = 1$
$U_{l,1,c}{}'[t]$
$= \text{prf} * \lambda_{l,1,c} * X_{l,1,c}[t] - (\mu_{1,c} + d_{1,c} + \theta + \Delta_1) * U_{l,1,c}[t]$

$i = 2^+$
$U_{l,i,c}{}'[t]$
$= d_{i-1,c} * U_{l,i-1,c}[t] + \text{prf} * \lambda_{l,i,c} * X_{l,i,c}[t] - (\mu_{i,c} + d_{i,c} + \theta + \Delta_i) * U_{l,i,c}[t]$

Recovered persons who did not seroconvert: ZS

$ZS_{l,1,c}{}'[t]$
$= (1 - \iota_{1,c}) * (\gamma_{1,c} * Y_{l,1,c}[t]) - ((1 - \psi zs_{1,c}) * \lambda_{l,1,c} + \phi c_{l,1,c} + \sigma zs_{1,c} + \theta szs_{1,c} + \mu_{1,c} + d_{1,c} + \Delta_1) * ZS_{l,1,c}[t]$

$ZS_{l,i,c}{}'[t]$
$= d_{i-1,c} * ZS_{l,i-1,c}[t] + (1 - \iota_{i,c}) * (\gamma_{i,c} * Y_{l,i,c}[t])$
$-((1 - \psi zs_{i,c}) * \lambda_{l,i,c} + \phi c_{l,i,c} + \sigma zs_{i,c} + \theta szs_{i,c} + \mu_{i,c} + d_{i,c} + \Delta_i) * ZS_{l,i,c}[t]$

Recovered persons who seroconverted: Z

$Z_{l,1,c}{}'[t]$
$= \iota_{1,c} * (\gamma_{1,c} * Y_{l,1,c}[t]) - ((1 - \psi z_{1,c}) * \lambda_{l,1,c} + \phi c_{l,1,c} + \sigma z_{1,c} + \theta s z_{1,c} + \mu_{1,c} + d_{1,c} + \Delta_1) * Z_{l,1,c}[t]$

$Z_{l,i,c}{}'(t)$
$= d_{i-1,c} * Z_{l,i-1,c}[t] + \iota_{i,c} * (\gamma_{i,c} * Y_{l,i,c}[t])$
$-((1 - \psi z_{i,c}) * \lambda_{l,i,c} + \phi c_{l,i,c} + \sigma z_{i,c} + \theta s z_{i,c} + \mu_{i,c} + d_{i,c} + \Delta_i) * Z_{l,i,c}[t]$

Infectious vaccinated persons: WS

$WS_{l,1,c}{}'[t]$

$= (1-\text{prf}) * (\lambda_{l,1,c} * VS_{l,1,c}[t]) + ((1-\psi q_{1,c}) * \lambda_{l,1,c} + \theta sq_{1,c}) * Q_{l,1,c}[t]$
$+((1-\psi qs_{1,c}) * \lambda_{l,1,c} + \theta sqs_{1,c}) * QS_{l,1,c}[t] - (\mu_{1,c} + d_{1,c} + \alpha_2 * \gamma_{1,c} + \theta tw_s + \Delta_1) * WS_{l,1,c}[t]$

${WS_{l,i,c}}'[t]$
$= d_{i-1,c} * WS_{l,i-1,c}[t] + (1-\text{prf}) * (\lambda_{l,i,c} * VS_{l,i,c}[t]) + ((1-\psi q_{i,c}) * \lambda_{l,i,c} + \theta sq_{i,c}) * Q_{l,i,c}[t]$
$+((1-\psi qs_{i,c}) * \lambda_{l,i,c} + \theta sqs_{i,c}) * QS_{l,i,c}[t] - (\mu_{i,c} + d_{i,c} + \alpha_2 * \gamma_{i,c} + \theta tw_s + \Delta_i) * WS_{l,i,c}[t]$

Infectious vaccinated persons who had one dose: W1
${W1_{l,1,c}}'[t]$
$= (1-\text{prf}) * ((1-\psi v_c^I) * \lambda_{l,1,c} * V1_{l,1,c}[t]) - (\mu_{1,c} + d_{1,c} + \alpha_2 * \gamma_{1,c} + \theta tw_1 + \Delta_1) * W1_{l,1,c}[t]$

${W1_{l,i,c}}'[t]$
$= d_{i-1,c} * W1_{l,i-1,c}[t] + (1-\text{prf}) * ((1-\psi v_c^I) * \lambda_{l,i,c} * V1_{l,i,c}[t])$
$-(\mu_{i,c} + d_{i,c} + \alpha_2 * \gamma_{i,c} + \theta tw_1 + \Delta_i) * W1_{l,i,c}[t]$

Infectious vaccinated persons who had two dose: W2
${W2_{l,i,c}}'[t]$
$= (1-\text{prf}) * ((1-\psi v_c^{II}) * \lambda_{l,1,c} * V2_{l,1,c}[t]) - (\mu_{1,c} + d_{1,c} + \alpha_2 * \gamma_{1,c} + \theta tw_2 + \Delta_1) * W2_{l,1,c}[t]$

${W2_{l,i,c}}'[t]$
$= d_{i-1,c} * W2_{l,i-1,c}[t] + (1-\text{prf}) * ((1-\psi v_c^{II}) * \lambda_{l,i,c} * V2_{l,i,c}[t])$
$-(\mu_{i,c} + d_{i,c} + \alpha_2 * \gamma_{i,c} + \theta tw_2 + \Delta_i) * W2_{l,i,c}[t]$

Persistently Infected vaccinated persons: PS
${PS_{l,1,c}}'[t]$
$= \text{prf} * (\lambda_{l,1,c} * VS_{l,1,c}[t]) - (\mu_{1,c} + d_{1,c} + \theta ps + \Delta_1) * PS_{l,1,c}[t]$

${PS_{l,i,c}}'[t]$
$= d_{i-1,c} * PS_{l,i-1,c}[t] + \text{prf} * (\lambda_{l,i,c} * VS_{l,i,c}[t]) - (\mu_{i,c} + d_{i,c} + \theta ps + \Delta_i) * PS_{l,i,c}[t]$

Persistently Infected vaccinated persons who had one dose: P1
${P1_{l,1,c}}'[t]$
$= \text{prf} * ((1-\psi p_{1,c}) * \lambda_{l,1,c} * V1_{l,1,c}[t]) - (\mu_{1,c} + d_{1,c} + \theta p_1 + \Delta_1) * P1_{l,1,c}[t]$

${P1_{l,i,c}}'[t]$
$= d_{i-1,c} * P1_{l,i-1,c}[t] + \text{prf} * ((1-\psi p_{1,c}) * \lambda_{l,i,c} * V1_{l,i,c}[t]) - (\mu_{i,c} + d_{i,c} + \theta p_1 + \Delta_i) * P1_{l,i,c}[t]$

Persistently Infected vaccinated persons who had two dose: P2

$P2_{l,1,c}{}^{'}[t]$
$= \text{prf} * ((1 - \psi p_{2,c}) * \lambda_{l,1,c} * V2_{l,1,c}[t]) - (\mu_{1,c} + d_{1,c} + \theta p_2 + \Delta_1 * P2_{l,1,c}[t]$

$P2_{l,i,c}{}^{'}[t]$
$= d_{i-1,c}[t] * P2_{l,i-1,c}[t] + \text{prf} * ((1 - \psi p_{2,c}) * \lambda_{l,i,c} * V2_{l,i,c}[t]) - (\mu_{i,c} + d_{i,c} + \theta p_2 + \Delta_i * P2_{l,i,c}[t]$

Recovered vaccinated persons with breakthroughs who did not seroconvert: QS

$QS_{l,1,c}{}^{'}[t]$
$= (1 - \iota_{1,c}) * \alpha_2 * \gamma_{1,c} * (WS_{l,1,c}[t] + W1_{l,1,c}[t] + W2_{l,1,c}[t]$
$-((1 - \psi qs_{1,c}) * \lambda_{l,1,c} + \sigma qs_{1,c} + \theta sqs_{1,c} + \mu_{1,c} + d_{1,c} + \Delta_1) * QS_{l,1,c}[t]$

$QS_{l,i,c}{}^{'}[t]$
$= d_{i-1,c} * QS_{l,i-1,c}[t] + (1 - \iota_{i,c}) * \alpha_2 * \gamma_{i,c} * (WS_{l,i,c}[t] + W1_{l,i,c}[t] + W2_{l,i,c}[t]$
$-((1 - \psi qs_{i,c}) * \lambda_{l,i,c} + \sigma qs_{i,c} + \theta sqs_{i,c} + \mu_{i,c} + d_{i,c} + \Delta_i) * QS_{l,i,c}[t]$

Recovered vaccinated persons with breakthroughs who seroconverted: Q

$Q_{l,1,c}{}^{'}[t]$
$= \iota_{1,c} * \alpha_2 * \gamma_{1,c} * (WS_{l,1,c}[t] + W1_{l,1,c}[t] + W2_{l,1,c}[t])$
$-((1 - \psi q_{1,c}) * \lambda_{l,1,c} + \sigma q_{1,c} + \theta sq_{1,c} + \mu_{1,c} + d_{1,c} + \Delta_1) * Q_{l,1,c}[t]$

$Q_{l,i,c}{}^{'}[t]$
$= d_{i-1,c} * Q_{l,i-1,c}[t] + \iota_{i,c} * \alpha_2 * \gamma_{i,c} * (WS_{l,i,c}[t] + W1_{l,i,c}[t] + W2_{l,i,c}[t])$
$-((1 - \psi q_{i,c}) * \lambda_{l,i,c} + \sigma q_{i,c} + \theta sq_{i,c} + \mu_{i,c} + d_{i,c} + \Delta_i) * Q_{l,i,c}[t]$

Tonsillectomy: Hx, Hy, Hz

$Hx_{l,1,c}{}^{'}[t]$
$$= \Delta_1 * (\sum_c X_{l,1,s}[t]) + \sigma z_{1,c} * Hz_{l,1}[t] + \sigma zs_{1,c} * Hzs_{l,1}[t] - (\lambda_{l,1,c} + \mu_{1,c} + d_{1,c}) * Hx_{l,1}[t]$$

$Hx_{l,i,c}{}^{'}[t]$
$$= d_{i-1,c} * Hx_{l,i-1}[t] + \Delta_i * (\sum_c X_{l,i,s}[t]) + \sigma z_{i,c} * Hz_{l,i}[t] + \sigma zs_{i,c} * Hzs_{l,i}[t] - (\lambda_{l,i,c} + \mu_{i,c} + d_{i,c}) * Hx_{l,i}[t]$$

$Hy_{l,1,c}{}^{'}[t]$
$= \lambda_{l,1,c} * (Hx_{l,1}[t] + Hvs_{l,1}[t]) + ((1 - \psi z_{1,c}) * \lambda_{l,1,c} + \theta sz_{1,c}) * Hz_{l,1}[t]$
$$+((1 - \psi zs_{1,c}) * \lambda_{l,1,c} + \theta szs_{1,c}) * Hzs_{l,1}[t] + \Delta_1 * \sum_c (Y_{l,1,c}[t] + U_{l,1,c}[t]) - (\mu_{1,c} + d_{1,c} + \gamma_{1,c}) * Hy_{l,1}[t]$$

$Hy_{l,i,c}{}^{'}[t]$
$= d_{i-1,c} * Hy_{l,i-1}[t] + \lambda_{l,i,c} * (Hx_{l,i}[t] + Hvs_{l,i}[t])$
$+((1 - \psi z_{i,c}) * \lambda_{l,i,c} + \theta sz_{i,c}) * Hz_{l,i}[t] + ((1 - \psi zs_{i,c}) * \lambda_{l,i,c} + \theta szs_{i,c}) * Hzs_{l,i}[t]$

$$+\Delta_i * \sum_c (Y_{l,i,c}[t] + U_{l,i,c}[t]) - (\mu_{i,c} + d_{i,c} + \gamma_{i,c}) * Hy_{l,i}[t]$$

$$Hzs_{l,1,c}{}'[t]$$
$$= (1 - \iota_{1,c}) * \gamma_{1,c} * Hy_{l,1}[t] + \Delta_1 * (\sum_c ZS_{l,1,c}[t])$$
$$-((1 - \psi zs_{1,c}) * \lambda_{l,1,c} + \sigma zs_{1,c} + \theta szs_{1,c} + \mu_{1,c} + d_{1,c}) * Hzs_{l,1}[t]$$

$$Hzs_{l,i,c}{}'[t]$$
$$= d_{i-1,c} * Hzs_{l,i-1}[t] + (1 - \iota_{i,c}) * \gamma_{i,c} * Hy_{l,i}[t] + \Delta_i * (\sum_c ZS_{l,i,c}[t])$$
$$-((1 - \psi zs_{i,c}) * \lambda_{l,i,c} + \sigma zs_{i,c} + \theta szs_{i,c} + \mu_{i,c} + d_{i,c}) * Hzs_{l,i}[t]$$

$$Hz_{l,1,c}{}'[t]$$
$$= \iota_{1,c} * \gamma_{1,c} * Hy_{l,1}[t] + \Delta_1 * (\sum_c Z_{l,1,c}[t]) - ((1 - \psi z_{1,c}) * \lambda_{l,1,c} + \sigma z_{1,c} + \theta sz_{1,c} + \mu_{1,c} + d_{1,c}) * Hz_{l,1}[t]$$

$$Hz_{l,i,c}{}'[t]$$
$$= d_{i-1,c} * Hz_{l,i-1}[t] + \iota_{i,c} * \gamma_{i,c} * Hy_{l,i}[t]$$
$$+\Delta_i * (\sum_c Z_{l,i,c}[t]) - ((1 - \psi z_{i,c}) * \lambda_{l,i,c} + \sigma z_{i,c} + \theta sz_{i,c} + \mu_{i,c} + d_{i,c}) * Hz_{l,i}[t]$$

Tonsillectomy: Hv, Hw, Hq

$$Hv1_{l,1,c}{}'[t]$$
$$= \Delta_1 * (\sum_c V1_{l,1,c}[t]) - ((1 - \psi v_c^I) * \lambda_{l,1,c} + \sigma v_{1,c}^I + \mu_{1,c} + d_{1,c}) * Hv1_{l,1}[t]$$

$$Hv1_{l,i,c}{}'[t]$$
$$= d_{i-1,c} * Hv1_{l,i-1}[t] + \Delta_i * (\sum_c V1_{l,i,c}[t]) - ((1 - \psi v_c^I) * \lambda_{l,i,c} + \sigma v_{i,c}^I + \mu_{i,c} + d_{i,c}) * Hv1_{l,i}[t]$$

$$Hv2_{l,1,c}{}'[t]$$
$$= \Delta_1 * (\sum_c V2_{l,1,c}[t]) - ((1 - \psi vc^{II}) * \lambda_{l,1,c} + \sigma v_{1,c}^{II} + \mu_{1,c} + d_{1,c}) * Hv2_{l,1}[t]$$

$$Hv2_{l,i,c}{}'[t]$$
$$= d_{i-1,c} * Hv2_{l,i-1}[t] + \Delta_i * (\sum_c V2_{l,i,c}[t]) - ((1 - \psi v_c^{II}) * \lambda_{l,i,c} + \sigma v_{i,c}^{II} + \mu_{i,c} + d_{i,c}) * Hv2_{l,i}[t]$$

$$Hvs_{l,1,c}{}'[t]$$
$$= \Delta_1 * (\sum_c VS_{l,1,c}[t]) + \sigma v_{1,c}^I * Hv1_{l,1}[t] + \sigma v_{1,c}^{II} * Hv2_{l,1}[t]$$
$$+\sigma q_{1,c} * Hq_{l,1}[t] + \sigma qs_{1,c} * Hqs_{l,1}[t] - (\lambda_{l,1,c} + d_{1,c} + \mu_{1,c}) * Hvs_{l,1}[t]$$

$$Hvs_{l,i,c}{}'[t]$$
$$= d_{i-1,c} * Hvs_{l,i-1}[t] + \Delta_i * (\sum_c VS_{l,i,c}[t]) + \sigma v_{i,c}^I * Hv1_{l,i}[t] + \sigma v_{i,c}^{II}[t] * Hv2_{l,i}[t]$$
$$+\sigma q_{i,c} * Hq_{l,i}[t] + \sigma qs_{i,c} * Hqs_{l,i}[t] - (\lambda_{l,i,c} + d_{i,c} + \mu_{i,c}) * Hvs_{l,i}[t]$$

$$Hw_{l,1,c}{}'[t]$$
$$= (1-\psi v_c^I) * \lambda_{l,1,c} * Hv1_{l,1}[t] + (1-\psi v_c^{II}) * \lambda_{l,1,c} * Hv2_{l,1}[t]$$
$$+((1-\psi q_{1,c}) * \lambda_{l,1,c} + \theta sq_{1,c}) * Hq_{l,1}[t] + ((1-\psi qs_{1,c}) * \lambda_{l,1,c} + \theta sqs_{1,c}) * Hqs_{l,1}[t]$$
$$+\Delta_1 * \sum_c (WS_{l,1,c}[t] + W1_{l,1,c}[t] + W2_{l,1,c}[t] + PS_{l,1,c}[t] + P1_{l,1,c}[t] + P2_{l,1,c}[t])$$
$$-(\mu_{1,c} + d_{1,c} + \alpha_2 * \gamma_{1,c}) * Hw_{l,1}[t]$$

$$Hw_{l,i,c}{}'[t]$$
$$= d_{i-1,c} * Hw_{l,i-1}[t] + (1-\psi v_c^I) * \lambda_{l,i,c} * Hv1_{l,i}[t] + (1-\psi v_c^{II}) * \lambda_{l,i,c} * Hv2_{l,i}[t]$$
$$+((1-\psi q_{i,c}) * \lambda_{l,i,c} + \theta sq_{i,c}) * Hq_{l,i}[t] + ((1-\psi qs_{i,c}) * \lambda_{l,i,c} + \theta sqs_{i,c}) * Hqs_{l,i}[t]$$
$$+\Delta_i * \sum_c (WS_{l,i,c}[t] + W1_{l,i,c}[t] + W2_{l,i,c}[t] + PS_{l,i,c}[t] + P1_{l,i,c}[t] + P2_{l,i,c}[t])$$
$$-(\mu_{i,c} + d_{i,c} + \alpha_2 * \gamma_{i,c}) * Hw_{l,i}[t]$$

$$Hqs_{l,1,c}{}'[t]$$
$$= (1-\iota_{1,c}) * \alpha_2 * \gamma_{1,c} * Hw_{l,1}[t] + \Delta_1 * (\sum_c QS_{l,1,c}[t])$$
$$-((1-\psi qs_{1,c}) * \lambda_{l,1,c} + \sigma qs_{1,c} + \theta sqs_{1,c} + \mu_{1,c} + d_{1,c}) * Hqs_{l,1}[t]$$

$$Hqs_{l,i,c}{}'[t]$$
$$= d_{i-1,c} * Hqs_{l,i-1}[t] + (1-\iota_{i,c}) * \alpha_2 * \gamma_{i,c} * Hw_{l,i}[t] + \Delta_i * (\sum_c QS_{l,i,c}[t])$$
$$-((1-\psi qs_{i,c}) * \lambda_{l,i,c} + \sigma qs_{i,c} + \theta sqs_{i,c} + \mu_{i,c} + d_{i,c}) * Hqs_{l,i}[t]$$

$$Hq_{l,1,c}{}'[t]$$
$$= \iota_{1,c} * \alpha_2 * \gamma_{1,c} * Hw_{l,1}[t] + \Delta_1 * (\sum_c Q_{l,1,c}[t])$$
$$-((1-\psi q_{1,c}) * \lambda_{l,1,c} + \sigma q_{1,c} + \theta sq_{1,c} + \mu_{1,c} + d_{1,c}) * Hq_{l,1}[t]$$

$$Hq_{l,i,c}{}'[t]$$
$$= d_{i-1,c} * Hq_{l,i-1}[t] + \iota_{i,c} * \alpha_2 * \gamma_{i,c} * Hw_{l,i}[t] + \Delta_i * (\sum_c Q_{l,i,c}[t])$$
$$-((1-\psi q_{i,c}) * \lambda_{l,i,c} + \sigma q_{i,c} + \theta sq_{i,c} + \mu_{i,c} + d_{i,c}) * Hq_{l,i}[t]$$

Detected oropharyngeal cancer

$$DOPCl_{l,1,c}{}'[t]$$
$$= p_L * \theta_{tL} * Y_{l,1,c}(t)$$
$$+p_L * (\theta_{p1} * P1_{l,1,c}(t) + \theta_{p2} * P2_{l,1,c}(t) + \theta_{ps} * PS_{l,1,c}(t))$$
$$+p_L * (\theta_{tw1} * W1_{l,1,c}(t) + \theta_{tw2} * W2_{l,1,c}(t) + \theta_{tws} * WS_{l,1,c}(t))$$
$$+p_L * \theta * U_{l,1,c} + p_L * \theta_{hw} * Hw_{l,1,c} + p_L * \theta_{hy} * Hy_{l,1,c}$$
$$-(\mu_{1,c} + d_{1,c} + \chi_{1,1} + \Omega_L + \theta_{tR}) * DOPCl_{l,1,c}[t]$$

$$DOPCl_{l,i,c}{}'[t]$$
$$= d_{i-1,c} * DOPCl_{l,i-1,c}[t] + p_L * \theta_{tL} * Y_{l,i,c}(t)$$
$$+p_L * (\theta_{p1} * P1_{l,i,c}(t) + \theta_{p2} * P2_{l,i,c}(t) + \theta_{ps} * PS_{l,i,c}(t))$$
$$+p_L * (\theta_{tw1} * W1_{l,i,c}(t) + \theta_{tw2} * W2_{l,i,c}(t) + \theta_{tws} * WS_{l,i,c}(t))$$
$$+p_L * \theta * U_{l,i,c} + p_L * \theta_{hw} * Hw_{l,i,c} + p_L * \theta_{hy} * Hy_{l,i,c}$$
$$-(\mu_{i,c} + d_{i,c} + \chi_{1,i} + \Omega_L + \theta_{tR}) * DOPCl_{l,i,c}[t]$$

$$DOPCr_{l,1,c}{}'[t]$$
$$= p_R * \theta_{tL} * Y_{l,1,c}(t) + p_R * \theta_{tR} * DOPCl_{l,1,c}$$
$$+p_R * (\theta_{p1} * P1_{l,1,c}(t) + \theta_{p2} * P2_{l,1,c}(t) + \theta_{ps} * PS_{l,1,c}(t))$$
$$+p_R * (\theta_{tw1} * W1_{l,1,c}(t) + \theta_{tw2} * W2_{l,1,c}(t) + \theta_{tws} * WS_{l,1,c}(t))$$
$$+p_R * \theta * U_{l,i,c} + p_L * \theta_{hw} * Hw_{l,1,c} + p_L * \theta_{hy} * Hy_{l,1,c}$$
$$-(\mu_{1,c} + d_{1,c} + \chi_{2,1} + \Omega_R + \theta_{tD}) * DOPCr_{l,1,c}[t]$$

$$DOPCr_{l,i,c}{}'[t]$$
$$= d_{i-1,c} * DOPCr_{l,i-1,c}[t] + p_R * \theta_{tR} * DOPCl_{l,i,c} + p_R * \theta_{tR} * Y_{l,i,c}(t)$$
$$+p_R * (\theta_{p1} * P1_{l,i,c}(t) + \theta_{p2} * P2_{l,i,c}(t) + \theta_{ps} * PS_{l,i,c}(t))$$
$$+p_R * (\theta_{tw1} * W1_{l,i,c}(t) + \theta_{tw2} * W2_{l,i,c}(t) + \theta_{tws} * WS_{l,i,c}(t))$$
$$+p_R * \theta * U_{l,i,c} + p_R * \theta_{hw} * Hw_{l,i,c} + p_R * \theta_{hy} * Hy_{l,i,c}$$
$$-(\mu_{i,c} + d_{i,c} + \chi_{2,i} + \Omega_R + \theta_{tD}) * DOPCr_{l,i,c}[t]$$

$$DOPCd_{l,1,c}{}'[t]$$
$$= p_D * \theta_{tL} * Y_{l,1,c}(t) + p_D * \theta_{tD} * DOPCr_{l,1,c}$$
$$+p_D * (\theta_{p1} * P1_{l,1,c}(t) + \theta_{p2} * P2_{l,1,c}(t) + \theta_{ps} * PS_{l,1,c}(t))$$
$$+p_D * (\theta_{tw1} * W1_{l,i,c}(t) + \theta_{tw2} * W2_{l,1,c}(t) + \theta_{tws} * WS_{l,1,c}(t))$$
$$+p_D * \theta * U_{l,1,c} + p_D * \theta_{hw} * Hw_{l,1,c} + p_D * \theta_{hy} * Hy_{l,1,c}$$
$$-(\mu_{1,c} + d_{1,c} + \chi_{3,1} + \Omega_D) * DOPCd_{l,1,c}[t]$$

$$DOPCd_{l,i,c}{}'[t]$$
$$= d_{i-1,c} * DOPCd_{l,i-1}[t] + p_D * \theta_{tD} * DOPCr_{l,i,c} + p_D * \theta_{tD} * Y_{l,i,c}(t)$$
$$+p_D * (\theta_{p1} * P1_{l,i,c}(t) + \theta_{p2} * P2_{l,i,c}(t) + \theta_{ps} * PS_{l,i,c}(t))$$
$$+p_D * (\theta_{tw1} * W1_{l,i,c}(t) + \theta_{tw2} * W2_{l,i,c}(t) + \theta_{tws} * WS_{l,i,c}(t))$$
$$+p_D * \theta * U_{l,i,c} + p_D * \theta_{hw} * Hw_{l,i,c} + p_D * \theta_{hy} * Hy_{l,i,c}$$
$$-(\mu_{i,c} + d_{i,c} + \chi_{3,i} + \Omega_D) * DOPCd_{l,i,c}[t]$$

Oropharyngeal cancer survivors

$$SOPC_{l,1,c}{}'[t]$$
$$= \Omega_L * DOPCl_{l,1,c}[t] + \Omega_R * DOPCr_{l,1,c}[t] + \Omega_D * DOPCd_{l,1,c}[t] - (\mu_{1,c} + d_{1,c}) * SOPC_{l,1,c}[t]$$

$$SOPC_{l,i,c}{}'[t]$$
$$= d_{i-1,c} * SOPC_{l,i-1,c}[t] + \Omega_L * DOPCl_{l,i,c}[t] + \Omega_R * DOPCr_{l,i,c}[t] + \Omega_D * DOPCd_{l,i,c}[t]$$
$$-(\mu_{i,c} + d_{i,c}) * SOPC_{l,i,c}[t]$$

$$N_{1,l,1}{}'[t]$$
$$= \sum_b B_{l,c} - (d_{1,c} + \mu_{1,c}) * N_{l,1,c}[t] - \chi_{1,1} * DOPCl_{l,1,c}[t] - \chi_{2,1} * DOPCr_{l,1,c}[t] - \chi_{3,1} * DOPCd_{l,1,c}[t]$$

$$N_{1,l,i}{}'[t]$$
$$= d_{i-1,c} * N_{1,l,i-1}[t] - (d_{i,c} + \mu_{i,c}) * N_{l,i,c}[t] - \chi_{1,i} * DOPCl_{l,i,c}[t] - \chi_{2,i} * DOPCr_{l,i,c}[t] - \chi_{3,i} * DOPCd_{l,i,c}[t]$$

Years of life

$$YL_a$$
$$= \int_0^T (\sum_l \sum_i \sum_c N_{l,i,c}) * e^{-\xi t} dt$$

Quality-adjusted life years

$$QALY_a$$
$$= \int_0^T e^{-\xi t} \sum_l \sum_i \sum_c q_{i,c} * (N_{l,i,c} - (1 - qopc_l) * SOPC_{l,i,c} - (1 - qopc_l) * DOPCl_{l,i,c}$$
$$-(1 - qopc_r) * DOPCr_{l,i,c} - (1 - qopc_d) * DOPCd_{l,i,c}) dt$$

Treatment costs

$$Treat(t)$$
$$=$$

Vaccination costs

$$Vaccinate(t)$$
$$= vaccine * \sum_l \sum_i \sum_c B_{l,c} * \phi_{l,c} + \phi c_{l,i,c} * (Y_{l,i,c} + X_{l,i,c} + U_{l,i,c} + Z_{l,i,c} + ZS_{l,i,c} + Hx_{l,i,c}$$
$$+Hy_{l,i,c} + Hz_{l,i,c} + Hzs_{l,i,c})$$
