# Supplementary material for: An economic and disease transmission model of human papillomavirus and oropharyngeal cancer in Texas
Source: Sci Rep. 2021 Jan 19;11:1802. doi: 10.1038/s41598-021-81375-5 (PMC7815750; doi:10.1038/s41598-021-81375-5)
Supplement: Supplementary file 3 — Supplementary Text S2. [file 41598_2021_81375_MOESM3_ESM.pdf]

Supplemental for: An Economic and Disease Transmission Model of Human Papillomavirus and Oropharyngeal Cancer in Texas

Chengxue Zhong, Li Xu, Ho-Lan Peng, Samantha Tam, Li Xu, Kristina R. Dahlstrom, Chi-Fang Wu, Shuangshuang Fu, Wenyaw Chan, Erich M. Sturgis, Lois M. Ramondetta, Libin Rong, David R. Lairson, Hongyu Miao

Text S2: Parameters and Initial Condition for Texas in Year 2010:

Rate of hysterectomy δ:

$i =$	0	1-8	9-10	11-12	13-14	15-17	18	19	20-24	25-26	27-29	30-34
	0.47	0.47	0.47	0.47	0.47	0.08	0.08	0.08	0.08	0.08	0.08	0.08
$i =$	35-39	40-44	45-49	50-54	55-59	60-64	65-69	70-74	75-79	80-84	85	
	0.08	0.08	0.08	0.08	0.08	0.08	0.08	0.08	0.08	0.08	0.08	

Transfer rate from age group i: d

$i =$	0	1-8	9-10	11-12	13-14	15-17	18	19	20-24	25-26	27-29	30-34
male	0.9966	0.1249	0.4999	0.4999	0.4999	0.3328	0.9995	0.9995	0.1995	0.4993	0.3327	0.1993
female	0.9971	0.1248	0.4999	0.4999	0.4999	0.3331	0.9998	0.9998	0.1998	0.4997	0.3330	0.1997
$i =$	35-39	40-44	45-49	50-54	55-59	60-64	65-69	70-74	75-79	80-84	85	
	0.1989	0.1989	0.1975	0.1975	0.1944	0.1944	0.1885	0.1885	0.1719	0.1719	0.0164	
	0.1994	0.1994	0.1945	0.1945	0.1850	0.1850	0.1816	0.1816	0.1292	0.1292	0.0003	

Death rate μ:

$i =$	0	1-8	9-10	11-12	13-14	15-17	18	19	20-24	25-26	27-29	30-34
male	6.71E-03	2.23E-04	1.44E-04	1.44E-04	1.44E-04	9.83E-04	9.83E-04	9.83E-04	9.83E-04	1.33E-03	1.33E-03	1.33E-03
female	5.74E-03	3.78E-04	1.15E-04	1.15E-04	1.15E-04	4.06E-04	4.06E-04	4.06E-04	4.06E-04	6.47E-04	6.47E-04	6.47E-04
$i =$	35-39	40-44	45-49	50-54	55-59	60-64	65-69	70-74	75-79	80-84	85	
	2.10E-03	2.10E-03	5.07E-03	5.07E-03	1.13E-02	1.13E-02	2.36E-02	2.36E-02	5.90E-02	5.90E-02	1.57E-01	
	1.24E-03	1.24E-03	3.25E-03	3.25E-03	6.75E-03	6.75E-03	1.56E-02	1.56E-02	4.36E-02	4.36E-02	1.38E-01	

Relative partner acquisition rate for sexual activity group pc_l:

$l =$	1	2	3
	1	2.96	11.29

Relative partner acquisition rate for age group pa_i:

$i =$	0	1-8	9-10	11-12	13-14	15-17	18	19	20-24	25-26	27-29	30-34
	0	0	0	0.055	0.11	1.18	2.42	2.42	2.61	2.55	2.55	1.72
$i =$	35-39	40-44	45-49	50-54	55-59	60-64	65-69	70-74	75-79	80-84	85	
	1.65	1.53	1.38	1.25	1.00	0.61	0.61	0.44	0.44	0.44	0.44	

Mean partner acquisition rate $\bar{c}_j$:

$i =$	0	1-8	9-10	11-12	13-14	15-17	18	19	20-24	25-26	27-29	30-34
	0	0	0	0.05	0.10	0.30	1.30	1.30	1.30	1.30	1.30	1.30
$i =$	35-39	40-44	45-49	50-54	55-59	60-64	65-69	70-74	75-79	80-84	85	
	1.30	1.30	1.30	1.30	1.30	0.50	0.50	0.50	0.50	0.50	0.50	

Rate of local oropharyngeal cancer-associated death (L:1, R:2, D:3) χ:

$i =$	0	1-8	9-10	11-12	13-14	15-17	18	19	20-24	25-26	27-29	30-34
Local	NA	NA	NA	NA	NA	0	0	0	0	0	0	0.0252
Regional	0	1	1	1	1	0	0	0	0.1532	0.172	0.172	0.1191
Distance	NA	NA	1	1	1	1	1	1	0.375	0.4647	0.4647	0.175
$i =$	35-39	40-44	45-49	50-54	55-59	60-64	65-69	70-74	75-79	80-84	85	
	0.0333	0.0251	0.0362	0.0386	0.0386	0.0505	0.061	0.0956	0.1314	0.0166	0.276	
	0.0803	0.0779	0.0849	0.0893	0.0942	0.11	0.139	0.1972	0.2532	0.3418	0.5011	
	0.2186	0.2483	0.2367	0.2384	0.2542	0.2473	0.2958	0.3885	0.4477	0.4968	0.6634	

Force of infection : λ

male:

$i =$	0	1-8	9-10	11-12	13-14	15-17	18	19	20-24	25-26	27-29	30-34
l=1	0	0	0	4.50E-01	1.00E-04	4.00E-04	3.10E-03	4.00E-03	4.50E-03	4.00E-03	4.60E-03	2.70E-03
l=2	0	0	0	0	1.00E-03	2.30E-03	1.26E-02	1.13E-02	1.14E-02	1.21E-02	1.52E-02	1.08E-02
l=3	0	0	0	0	6.10E-03	1.17E-02	9.87E-02	6.25E-02	7.43E-02	8.32E-02	8.38E-02	5.47E-02
$i =$	35-39	40-44	45-49	50-54	55-59	60-64	65-69	70-74	75-79	80-84	85	
l=1	2.40E-03	2.10E-03	1.80E-03	1.60E-03	1.30E-03	1.20E-03	1.40E-03	1.00E-03	1.00E-03	1.00E-03	1.10E-03	
l=2	9.40E-03	8.90E-03	7.80E-03	6.90E-03	5.30E-03	4.80E-03	5.20E-03	3.60E-03	3.60E-03	3.70E-03	4.00E-03	
l=3	5.09E-02	7.71E-02	6.62E-02	5.78E-02	4.36E-02	3.51E-02	3.78E-02	2.46E-02	2.43E-02	2.45E-02	2.61E-02	

female:

$i =$	0	1-8	9-10	11-12	13-14	15-17	18	19	20-24	25-26	27-29	30-34
l=1	0	0	0	7.76E-01	2.00E-04	8.00E-04	5.90E-03	6.70E-03	7.40E-03	6.70E-03	6.90E-03	4.80E-03
l=2	0	0	0	0	1.20E-03	2.70E-03	1.65E-02	1.83E-02	1.84E-02	2.34E-02	2.32E-02	1.62E-02
l=3	0	0	0	0	1.18E-02	2.46E-02	6.58E-02	8.19E-02	9.37E-02	1.44E-01	1.81E-01	1.06E-01
$i =$	35-39	40-44	45-49	50-54	55-59	60-64	65-69	70-74	75-79	80-84	85	
l=1	4.70E-03	4.20E-03	3.90E-03	3.50E-03	2.70E-03	2.70E-03	2.60E-03	1.80E-03	1.70E-03	1.50E-03	1.30E-03	
l=2	1.74E-02	1.70E-02	1.58E-02	1.42E-02	1.09E-02	1.04E-02	1.02E-02	6.80E-03	6.40E-03	6.00E-03	5.10E-03	
l=3	1.68E-01	1.13E-01	1.05E-01	9.34E-02	7.10E-02	6.19E-02	6.05E-02	3.92E-02	3.72E-02	3.49E-02	3.08E-02	

Annual growth rate: q

$i =$	0	1-8	9-10	11-12	13-14	15-17	18	19	20-24	25-26	27-29	30-34
male	0	0	0	0	0	0	0	0	0	0	0	0
female	0	0	0	0	0	0	0	0	0	0	0	0
$i =$	35-39	40-44	45-49	50-54	55-59	60-64	65-69	70-74	75-79	80-84	85	
	0	0	0	0	0	0	0	0	0	0	0	
	0	0	7.79e-03	7.79e-03	2.41e-02	2.41e-02	2.25e-02	2.25e-02	1.20e-01	1.20e-01	3.49e-01	

New borne: B

$c =$	1	2
l=1	186357	193489
l=2	0	0
l=3	0	0

Vaccine uptake rate with first dose, male persons : ϕ_{cm}

$i =$	19	20-24	25-26	else
l=1	0.003	0.006	0.006	0
l=2	0.003	0.006	0.006	0
l=3	0.003	0.006	0.006	0

Vaccine uptake rate with first dose, female persons : ϕ_{cf}

$i =$	13-14	15-17	18	19	20-24	25-26	else
l=1	0.475	0.475	0.207	0.207	0.207	0.207	0
l=2	0.475	0.475	0.207	0.207	0.207	0.207	0
l=3	0.475	0.475	0.207	0.207	0.207	0.207	0

Rate of progression

From HPV infection to local OPC: θt_L

$i =$	0	1-8	9-10	11-12	13-14	15-17	18	19	20-24	25-26	27-29	30-34
male	0	0	0	0	0	0	0	0	0	0.15	0.15	0.30
female	0	0	0	0	0	0	0	0	0	0.06	0.06	0.06
$i =$	35-39	40-44	45-49	50-54	55-59	60-64	65-69	70-74	75-79	80-84	85	
	0.40	0.40	0.50	0.50	0.50	0.50	0.50	0.50	0.50	0.50	0.50	
	0.06	0.06	0.06	0.06	0.08	0.08	0.18	0.20	0.18	0.15	0.15	

From local OPC to regional OPC: θt_R

$i =$	0	1-8	9-10	11-12	13-14	15-17	18	19	20-24	25-26	27-29	30-34
male	0	0	0	0	0	0	0	0	0	0.80	0.80	0.80
female	0	0	0	0	0	0	0	0	0	0.80	0.80	0.80
$i =$	35-39	40-44	45-49	50-54	55-59	60-64	65-69	70-74	75-79	80-84	85	
	0.80	0.80	0.80	0.80	0.96	0.96	0.96	0.96	0.96	0.96	0.96	
	0.80	0.80	0.80	0.80	0.96	0.96	0.96	0.96	0.96	0.96	0.96	

From regional OPC to distant OPC: θt_D

$i =$	0	1-8	9-10	11-12	13-14	15-17	18	19	20-24	25-26	27-29	30-34
male	0	0	0	0	0	0	0	0	0	0.64	0.64	0.64
female	0	0	0	0	0	0	0	0	0	0.64	0.64	0.64
$i =$	35-39	40-44	45-49	50-54	55-59	60-64	65-69	70-74	75-79	80-84	85	
	0.64	0.64	0.64	0.64	0.768	0.768	0.768	0.768	0.768	0.768	0.768	
	0.64	0.64	0.64	0.64	0.768	0.768	0.768	0.768	0.768	0.768	0.768	

Rate of recovery from HPV infection: $\gamma = 0.83$
Reactivation rate following sero-conversion for male: $\theta sz = 0.006$
Reactivation rate following sero-conversion for female: $\theta sz = 0.002$
Reactivation rate for male, who did not sero-convert: $\theta szs = 0.006$
Reactivation rate for female, who did not sero-convert: $\theta szs = 0.002$
Probability of sero-conversion following HPV clearance: $\iota = 0$
Degree of protection following sero-conversion for male: $\psi z = 0.5$
Degree of protection following sero-conversion for female: $\psi z = 0.8$
Degree of protection following no sero-conversion: $\psi zs = 0$
Rate of waning immunity following 1 dose vaccination: $\sigma v_1 = 0$

Rate of waning immunity following 2 dose vaccination: $\sigma v_2 = 0$

Rate of waning immunity following recovery with sero-conversion: $\sigma q = 0$

Rate of waning immunity following recovery without sero-conversion: $\sigma qs = 0$

Degree of protection following recovery of an infection in previously vaccinated individuals with sero-conversion: $\psi q = 100$

Degree of protection following recovery of an infection in previously vaccinated individuals without seroconversion: $\psi qs = 0$

Reactivation rate in patients who are recovered, vaccinated and seroconverted: $\theta sq = 0$

Reactivation rate in patients who are recovered, vaccinated and no seroconverted, male: $\theta sqs = 0.006$

Reactivation rate in patients who are recovered, vaccinated and no seroconverted, female: $\theta sqs = 0.002$

Proportion of infections that are destined to be persistent: prf=0.17

Degree of protection following sero-conversion, vaccinated with 1 dose: $\psi p_1 = 1.0$

Degree of protection following sero-conversion, vaccinated with 2 dose: $\psi p_2 = 1.0$

Proportion receiving only 1 dose: $\Phi_1 = 1$

Proportion receiving only 2 dose for male: $\Phi_2 m = 0.7229$

Proportion receiving only 2 dose for female: $\Phi_2 f = 0.8312$

Degree of protection with 1 dose: $\psi v_1 = 0.91$

Degree of protection with 2 dose: $\psi v_2 = 0.99$

Relative rate of recovery from breakthrough infection: $\alpha = 1.0$

Cure rate of local oropharyngeal cancer(L:1, R:2, D:3): $\Omega = 0$

Rate of progression from breakthrough infection to DOPC (with 1 dose): $\theta p_1 = 0$

Rate of progression from breakthrough infection to DOPC (with 2 dose): $\theta p_2 = 0$

Rate of progression to DOPC in patients that are persistently infected and vaccinated: $\theta p_s = 0$

Proportion of new borne vaccinated, male persons: $\phi m = 0$

Proportion of new borne vaccinated, female persons: $\phi f = 0$

Proportion of new born vaccinated: $\phi = 0$

Rate of waning immunity following recovery with sero-conversion: $\sigma z = 0$

Rate of waning immunity following recovery without sero-conversion: $\sigma zs = 0$

Rate of progression to DOPC in patients that are vaccinated with 1 dose, then are infected: $\theta tw_1 = 0$

Rate of progression to DOPC in patients that are vaccinated with 2 dose, then are infected: $\theta tw_2 = 0$

Rate of progression to DOPC in patients that are infected, vaccinated and have waning immunity: $\theta tw_s = 0$

Detection rate of local oropharyngeal cancer:$pL = 0.68$

Detection rate of regional oropharyngeal cancer: $pR = 0.85$

Detection rate of distant oropharyngeal cancer: $pD = 1.0$

Rate of progression from breakthrough infection to DOPC (with 1 dose): $\theta p_1 = 0$

Rate of progression from breakthrough infection to DOPC (with 2 dose): $\theta p_2 = 0$

Rate of progression to DOPC in patients that are persistently infected and vaccinated: $\theta p_s = 0$

Rate of progression from HPV infection (persistent) to DOPC in patients with tonsillectomy that are infected for male: $\theta hy = 0.8$

Rate of progression from HPV infection (persistent) to DOPC in patients with tonsillectomy that are infected for female: $\theta hy = 0.6$

Rate of progression from HPV infection (persistent) to DOPC in patients with tonsillectomy that are infected vaccinated for male: $\theta hw = 0.8$

Rate of progression from HPV infection (persistent) to DOPC in patients with tonsillectomy that are infected vaccinated for female: $\theta hw = 0.6$

Initial condition of Texas, 2010:

Persistently infected: U

female:

$i=$	0	1-8	9-10	11-12	13-14	15-17	18	19	20-24	25-26	27-29	30-34
l=1	0	0	0	0	0	1.20e+03	3.67e+02	3.55e+02	1.67e+03	7.74e+02	1.19e+03	1.91e+03
l=2	0	0	0	0	0	1.01e+02	0.52e+02	0.59e+02	3.50e+02	0.73e+02	1.17e+02	1.61e+02
l=3	0	0	0	0	0	0.57e+01	0.19e+02	0.21e+02	0.85e+02	0.11e+02	0.12e+02	0.22e+02

$i=$	35-39	40-44	45-49	50-54	55-59	60-64	65-69	70-74	75-79	80-84	85
l=1	1.97e+03	1.91e+03	1.99e+03	1.90e+03	1.65e+03	1.37e+03	1.01e+03	7.54e+02	6.04e+02	4.68e+02	4.59e+02
l=2	1.37e+02	1.01e+02	1.06e+02	1.01e+02	0.88e+02	0.72e+02	0.54e+02	0.40e+02	0.32e+02	0.25e+02	0.24e+02
l=3	0.76e+01	0.97e+01	0.10e+02	0.97e+01	0.83e+01	0.69e+01	0.51e+01	0.38e+01	0.31e+01	0.24e+01	0.23e+01

male:

$i=$	0	1-8	9-10	11-12	13-14	15-17	18	19	20-24	25-26	27-29	30-34
l=1	0	0	0	0	0	2.15e+03	6.90e+02	5.91e+02	2.75e+03	1.23e+03	1.87e+03	3.09e+03
l=2	0	0	0	0	0	0.91e+02	0.65e+02	1.20e+02	7.13e+02	1.94e+02	2.56e+02	2.58e+02
l=3	0	0	0	0	0	0.24e+02	0.11e+02	0.44e+02	1.78e+02	0.39e+02	0.83e+02	1.01e+02

$i=$	35-39	40-44	45-49	50-54	55-59	60-64	65-69	70-74	75-79	80-84	85
l=1	3.07e+03	3.08e+03	3.18e+03	3.01e+03	2.51e+03	2.06e+03	1.47e+03	1.03e+03	7.58e+02	5.05e+02	3.67e+02
l=2	2.74e+02	2.08e+02	2.15e+02	2.03e+02	1.70e+02	1.39e+02	0.99e+02	0.70e+02	0.51e+02	0.34e+02	0.25e+02
l=3	0.81e+02	0.24e+02	0.25e+02	0.23e+02	1.96e+01	6.05e+01	1.14e+01	0.81e+01	0.60e+01	0.39e+01	0.29e+01

Persistently infected vaccinated : PS

female:

$i=$	0	1-8	9-10	11-12	13-14	15-17	18	19	20-24	25-26	27-29	30-34
l=1	0	0	0	0	0	1.91e+03	2.54e+02	2.46e+02	1.16e+03	5.37e+02	0	0
l=2	0	0	0	0	0	1.62e+02	0.36e+02	0.41e+02	2.43e+02	0.51e+02	0	0
l=3	0	0	0	0	0	0.92e+01	0.13e+02	0.14e+02	0.59e+02	0.75e+01	0	0

$i=$	35-39	40-44	45-49	50-54	55-59	60-64	65-69	70-74	75-79	80-84	85
l=1	0	0	0	0	0	0	0	0	0	0	0
l=2	0	0	0	0	0	0	0	0	0	0	0
l=3	0	0	0	0	0	0	0	0	0	0	0

male:

$i=$	0	1-8	9-10	11-12	13-14	15-17	18	19	20-24	25-26	27-29	30-34
l=1	0	0	0	0	0	0	0	0.36e+01	0.34e+02	0.15e+02	0	0
l=2	0	0	0	0	0	0	0	0.07e+01	0.88e+01	0.24e+01	0	0
l=3	0	0	0	0	0	0	0	0.03e+01	0.22e+01	0.05e+01	0	0

$i=$	35-39	40-44	45-49	50-54	55-59	60-64	65-69	70-74	75-79	80-84	85
l=1	0	0	0	0	0	0	0	0	0	0	0
l=2	0	0	0	0	0	0	0	0	0	0	0
l=3	0	0	0	0	0	0	0	0	0	0	0

Persistently infected vaccinated with 1 dose : P1

female:

i =	0	1-8	9-10	11-12	13-14	15-17	18	19	20-24	25-26	27-29	30-34
l=1	0	0	0	0	0	6.34e+02	0.86e+02	0.83e+02	0.39e+03	1.81e+02	0	0
l=2	0	0	0	0	0	0.54e+02	0.12e+02	0.14e+02	0.82e+02	0.17e+02	0	0
l=3	0	0	0	0	0	0.30e+01	0.44e+01	0.49e+01	1.99e+01	0.25e+01	0	0

i =	35-39	40-44	45-49	50-54	55-59	60-64	65-69	70-74	75-79	80-84	85
l=1	0	0	0	0	0	0	0	0	0	0	0
l=2	0	0	0	0	0	0	0	0	0	0	0
l=3	0	0	0	0	0	0	0	0	0	0	0

male:

i =	0	1-8	9-10	11-12	13-14	15-17	18	19	20-24	25-26	27-29	30-34
l=1	0	0	0	0	0	0	0	0.11e+01	0.12e+02	0.52e+01	0	0
l=2	0	0	0	0	0	0	0	0.02e+01	0.30e+01	0.08e+01	0	0
l=3	0	0	0	0	0	0	0	0.083737	0.760011	0.164901	0	0

i =	35-39	40-44	45-49	50-54	55-59	60-64	65-69	70-74	75-79	80-84	85
l=1	0	0	0	0	0	0	0	0	0	0	0
l=2	0	0	0	0	0	0	0	0	0	0	0
l=3	0	0	0	0	0	0	0	0	0	0	0

Persistently infected vaccinated with 2 dose : P2

female:

i =	0	1-8	9-10	11-12	13-14	15-17	18	19	20-24	25-26	27-29	30-34
l=1	0	0	0	0	0	1.28e+03	1.69e+02	1.63e+02	7.68e+02	3.56e+02	0	0
l=2	0	0	0	0	0	1.08e+02	0.24e+02	0.27e+02	1.61e+02	0.34e+02	0	0
l=3	0	0	0	0	0	0.61e+01	0.87e+01	0.96e+01	0.39e+02	0.49e+01	0	0

i =	35-39	40-44	45-49	50-54	55-59	60-64	65-69	70-74	75-79	80-84	85
l=1	0	0	0	0	0	0	0	0	0	0	0
l=2	0	0	0	0	0	0	0	0	0	0	0
l=3	0	0	0	0	0	0	0	0	0	0	0

male:

i =	0	1-8	9-10	11-12	13-14	15-17	18	19	20-24	25-26	27-29	30-34
l=1	0	0	0	0	0	0	0	0.25e+01	0.22e+02	0.99e+01	0	0
l=2	0	0	0	0	0	0	0	0.05e+01	0.58e+01	0.16e+01	0	0
l=3	0	0	0	0	0	0	0	0.188409	0.14e+01	0.03e+01	0	0

i =	35-39	40-44	45-49	50-54	55-59	60-64	65-69	70-74	75-79	80-84	85
l=1	0	0	0	0	0	0	0	0	0	0	0
l=2	0	0	0	0	0	0	0	0	0	0	0
l=3	0	0	0	0	0	0	0	0	0	0	0

Population with tonsillectomy: Hx

female:

i =	0	1-8	9-10	11-12	13-14	15-17	18	19	20-24	25-26	27-29	30-34
l=1	8.76e+02	7.11e+03	1.78e+03	1.72e+03	1.68e+03	4.02e+02	1.23e+02	1.19e+03	5.61e+02	2.60e+02	3.96e+02	6.40e+02
l=2	0	0	0	0	0.29e+02	0.34e+02	0.17e+02	0.20e+02	1.18e+02	0.25e+02	0.39e+02	0.54e+02
l=3	0	0	0	0	0.17e+01	0.19e+01	0.63e+01	0.70e+01	0.29e+02	0.36e+01	0.39e+01	0.74e+01
i =	35-39	40-44	45-49	50-54	55-59	60-64	65-69	70-74	75-79	80-84	85	
	6.61e+02	6.41e+02	6.69e+02	6.40e+02	5.53e+02	4.60e+02	3.40e+02	2.53e+02	2.03e+02	1.57e+02	1.54e+02	
	0.46e+02	0.34e+02	0.36e+02	0.34e+02	0.29e+02	0.24e+02	0.18e+02	0.13e+02	0.11e+02	0.84e+01	0.82e+01	
	0.25e+01	0.32e+01	0.34e+01	0.32e+01	0.28e+01	0.23e+01	0.17e+01	0.13e+01	0.10e+01	0.797856	0.782644	

male:

i =	0	1-8	9-10	11-12	13-14	15-17	18	19	20-24	25-26	27-29	30-34
l=1	9.09e+02	7.41e+03	1.86e+03	1.81e+03	1.76e+03	4.40e+02	1.41e+02	1.21e+02	5.63e+02	2.52e+02	3.83e+02	6.33e+02
l=2	0	0	0	0	0.19e+02	0.19e+02	0.13e+02	0.25e+02	1.46e+02	0.40e+02	0.52e+02	0.53e+02
l=3	0	0	0	0	0.52e+01	0.50e+01	0.23e+01	0.90e+01	0.36e+02	0.79e+01	0.17e+02	0.21e+02
i =	35-39	40-44	45-49	50-54	55-59	60-64	65-69	70-74	75-79	80-84	85	
	6.28e+02	6.30e+02	6.50e+02	6.16e+02	5.14e+02	4.21e+02	3.00e+02	2.11e+02	1.55e+02	1.03e+02	0.75e+02	
	0.56e+02	0.43e+02	0.44e+02	0.42e+02	0.35e+02	0.28e+02	0.20e+02	0.14e+02	0.10e+02	0.70e+01	0.51e+01	
	0.17e+02	0.49e+01	0.51e+01	0.48e+01	0.40e+01	0.33e+01	0.23e+01	0.16e+01	0.12e+01	0.807002	0.586092	

Population of tonsillectomy that are infected: Hy

female:

i =	0	1-8	9-10	11-12	13-14	15-17	18	19	20-24	25-26	27-29	30-34
l=1	0	0	0	0	0	0.56e+01	0.17e+01	0.17e+01	0.79e+01	0.36e+01	0.56e+01	0.90e+01
l=2	0	0	0	0	0	0.05e-01	0.02e-01	0.03e-01	0.16e+01	0.03e-01	0.05e-01	0.08e-01
l=3	0	0	0	0	0	2.70e-02	8.95e-02	9.84e-02	0.04e-01	0.51e-01	0.55e-01	0.01e-01
i =	35-39	40-44	45-49	50-54	55-59	60-64	65-69	70-74	75-79	80-84	85	
	0.93e+01	0.90e+01	0.94e+01	0.90e+01	0.77e+01	0.64e+01	0.48e+01	0.35e+01	0.28e+01	0.22e+01	0.22e+01	
	0.06e-01	0.05e-01	0.05e-01	0.05e-01	0.04e-01	0.03e-01	0.03e-01	0.02e-01	0.02e-01	0.01e-01	0.01e-01	
	0.36e-01	0.45e-01	0.48e-01	0.45e-01	0.39e-01	0.33e-01	0.24e-01	0.18e-01	0.14e-01	0.11e-01	0.11e-01	

male:

i =	0	1-8	9-10	11-12	13-14	15-17	18	19	20-24	25-26	27-29	30-34
l=1	0	0	0	0	0	0.10e+02	0.32e+01	0.28e+01	0.13e+02	0.58e+01	0.88e+01	0.15e+02
l=2	0	0	0	0	0	0.043e+01	0.031e+01	0.06e+01	0.34e+01	0.09e+01	0.12e+01	0.12e+01
l=3	0	0	0	0	0	0.01e+01	0.53e-01	0.02e+01	0.08e+01	0.02e+01	0.04e+01	0.05e+01
i =	35-39	40-44	45-49	50-54	55-59	60-64	65-69	70-74	75-79	80-84	85	
	0.14e+02	0.14e+02	0.15e+02	0.14e+02	0.12e+02	0.97e+01	0.69e+01	0.49e+01	0.36e+01	0.24e+01	0.17e+01	
	0.13e+01	0.10e+01	0.10e+01	0.10e+01	0.08e+01	0.07e+01	0.05e+01	0.03e+01	0.02e+01	0.02e+01	0.01e+01	
	0.04e+01	0.01e+01	0.01e+01	0.01e+01	0.92e-01	0.76e-01	0.054	0.38e-01	0.28e-01	0.19e-01	0.13e-01	

Population with tonsillectomy that were infected, recovered, seroconverted: Hz

female:

$i =$	0	1-8	9-10	11-12	13-14	15-17	18	19	20-24	25-26	27-29	30-34
l=1	0	0	0	0	0	0.28e+02	0.852	0.824	0.39e+01	0.18e+01	0.28e+01	0.44e+01
l=2	0	0	0	0	0	0.02e+01	0.01e+01	0.01e+01	0.08e+01	0.02e+01	0.03e+01	0.04e+01
l=3	0	0	0	0	0	0.13e-01	0.44e-01	0.49e-01	0.02e-01	0.25e-01	0.27e-01	0.51e-01
$i =$	35-39	40-44	45-49	50-54	55-59	60-64	65-69	70-74	75-79	80-84	85	
	0.46e+01	0.44e+01	0.46e+01	0.44e+01	0.38e+01	0.32e+01	0.24e+01	0.18e+01	0.14e+01	0.11e+01	0.11e+01	
	0.03e+01	0.02e+01	0.02e+01	0.02e+01	0.02e+01	0.02e+01	0.01e+01	0.93e-01	0.75e-01	0.58e-01	0.58e-01	
	0.18e-01	0.22e-01	0.23e-01	0.22e-01	0.19e-01	0.16e-01	0.12e-01	0.89e-02	0.71e-02	0.55e-02	0.54e-02	

male:

$i =$	0	1-8	9-10	11-12	13-14	15-17	18	19	20-24	25-26	27-29	30-34
l=1	0	0	0	0	0	0.03e+01	0.97e-01	0.83e-01	0.04e+01	0.02e+01	0.03e+01	0.04e+01
l=2	0	0	0	0	0	0.13e-01	0.92e-02	0.17e-01	0.01e+01	0.27e-01	0.36e-01	0.36e-01
l=3	0	0	0	0	0	3.43e-03	1.58e-03	6.20e-03	2.50e-02	5.43e-03	1.17e-02	1.42e-02
$i =$	35-39	40-44	45-49	50-54	55-59	60-64	65-69	70-74	75-79	80-84	85	
	0.04e+01	0.04e+01	0.04e+01	0.04e+01	0.04e+01	0.03e+01	0.02e+01	0.01e+01	0.01e+01	0.71e-01	0.52e-01	
	0.39e-01	0.29e-01	0.30e-01	0.29e-01	0.24e-01	0.20e-01	0.14e-01	0.10e-01	0.72e-02	0.48e-02	0.35e-02	
	1.15e-02	3.38e-03	3.49e-03	3.30e-03	2.76e-03	2.26e-03	1.61e-03	1.13e-03	8.32e-04	5.54e-04	4.03e-04	

Population with tonsillectomy that were infected, recovered, not seroconverted: Hzs

female:

$i =$	0	1-8	9-10	11-12	13-14	15-17	18	19	20-24	25-26	27-29	30-34
l=1	0	0	0	0	0	0.19e+01	0.06e+01	0.06e+01	0.26e+01	0.12e+01	0.19e+01	0.30e+01
l=2	0	0	0	0	0	0.02e+01	0.83e+01	0.94e+01	0.55e+01	0.01e+01	0.02e+01	0.03e+01
l=3	0	0	0	0	0	0.91e-02	0.30e-01	0.33e-01	0.01e-01	0.17e-01	0.19e-01	0.35e-01
$i =$	35-39	40-44	45-49	50-54	55-59	60-64	65-69	70-74	75-79	80-84	85	
	0.31e+01	0.30e+01	0.32e+01	0.30e+01	0.26e+01	0.22e+01	0.16e+01	0.12e+01	0.10e+01	0.07e+01	0.07e+01	
	0.02e+01	0.02e+01	0.02e+01	0.02e+01	0.01e+01	0.01e+01	0.85e-01	0.63e-01	0.51e-01	0.39e-01	0.39e-01	
	0.12e-01	0.15e-01	0.16e-01	0.15e-01	0.13e-01	0.11e-01	0.81e-02	0.60e-02	0.48e-02	0.38e-02	0.37e-02	

male:

$i =$	0	1-8	9-10	11-12	13-14	15-17	18	19	20-24	25-26	27-29	30-34
l=1	0	0	0	0	0	8.10E+00	2.60E+00	2.23E+00	1.04E+01	4.63E+00	7.04E+00	1.16E+01
l=2	0	0	0	0	0	3.42E-01	2.46E-01	4.52E-01	2.69E+00	7.30E-01	9.63E-01	9.71E-01
l=3	0	0	0	0	0	9.18E-02	4.24E-02	1.66E-01	6.70E-01	1.45E-01	3.14E-01	3.81E-01
$i =$	35-39	40-44	45-49	50-54	55-59	60-64	65-69	70-74	75-79	80-84	85	
	1.16E+01	1.16E+01	1.20E+01	1.13E+01	9.46E+00	7.75E+00	5.52E+00	3.89E+00	2.85E+00	1.90E+00	1.38E+00	
	1.03E+00	7.84E-01	8.10E-01	7.66E-01	6.40E-01	5.24E-01	3.73E-01	2.63E-01	1.93E-01	1.29E-01	9.34E-02	
	3.07E-01	9.05E-02	9.35E-02	8.84E-02	7.38E-02	6.04E-02	4.31E-02	3.03E-02	2.23E-02	1.49E-02	1.08E-02	

Vaccinated with 1 dose, persons with tonsillectomy: Hv1

female:

$i =$	13-14	15-17	18	19	20-24	25-26	else
l=1	7.96E+02	1.91E+02	2.55E+01	2.47E+01	1.16E+02	5.39E+01	0
l=2	1.39E+01	1.62E+01	3.63E+00	4.13E+00	2.44E+01	5.10E+00	0
l=3	7.86E-01	9.16E-01	1.32E+00	1.45E+00	5.93E+00	7.48E-01	0

male:

$i =$	19	20-24	25-26	else
l=1	8.35E-03	7.78E-02	3.47E-02	0
l=2	1.69E-03	2.01E-02	5.48E-03	0
l=3	6.23E-04	5.03E-03	1.09E-03	0

Vaccinated with 2 dose, persons with tonsillectomy: Hv2

female:

$i =$	13-14	15-17	18	19	20-24	25-26	else
l=1	2.30E+02	5.51E+01	7.27E+00	7.03E+00	3.31E+01	1.54E+01	0
l=2	4.01E+00	4.67E+00	1.03E+00	1.18E+00	6.95E+00	1.45E+00	0
l=3	2.27E-01	2.64E-01	3.77E-01	4.14E-01	1.69E+00	2.13E-01	0

Vaccinated with 2 dose, persons with tonsillectomy for male: Hv2=0
Vaccinated with waned immunity, persons with tonsillectomy: Hvs=0

Infected vaccinated, persons with tonsillectomy: Hw

female:

$i =$	15-17	18	19	20-24	25-26	else
l=1	1.93E+01	2.58E+00	2.49E+00	1.17E+01	5.44E+00	0
l=2	1.64E+00	3.67E-01	4.17E-01	2.46E+00	5.15E-01	0
l=3	9.27E-02	1.34E-01	1.47E-01	5.99E-01	7.56E-02	0

male:

$i =$	19	20-24	25-26	else
l=1	6.56E-04	6.11E-03	2.73E-03	0
l=2	1.33E-04	1.58E-03	4.30E-04	0
l=3	4.90E-05	3.95E-04	8.57E-05	0

Recovered vaccinated without sero-convertion, persons with tonsillectomy: Hqs

female:

$i =$	15-17	18	19	20-24	25-26	else
l=1	5.99E+00	7.97E-01	7.71E-01	3.63E+00	1.68E+00	0
l=2	5.07E-01	1.13E-01	1.29E-01	7.62E-01	1.59E-01	0
l=3	2.87E-02	4.14E-02	4.54E-02	1.85E-01	2.34E-02	0

male:

$i =$	19	20-24	25-26	else
l=1	4.83E-04	4.50E-03	2.01E-03	0
l=2	9.81E-05	1.17E-03	3.17E-04	0
l=3	3.61E-05	2.91E-04	6.31E-05	0

Recovered vaccinated with sero-convertion, persons with tonsillectomy: Hq

female:

$i =$	15-17	18	19	20-24	25-26	else
l=1	8.80E+00	1.17E+00	1.13E+00	5.34E+00	2.47E+00	0
l=2	7.45E-01	1.67E-01	1.90E-01	1.12E+00	2.34E-01	0
l=3	4.22E-02	6.08E-02	6.68E-02	2.72E-01	3.44E-02	0

male:

$i =$	19	20-24	25-26	else
l=1	1.80E-05	1.68E-04	7.51E-05	0
l=2	3.66E-06	4.35E-05	1.18E-05	0
l=3	1.35E-06	1.09E-05	2.36E-06	0

Susceptible persons: X

female:

$i =$	0	1-8	9-10	11-12	13-14	15-17	18	19	20-24	25-26	27-29	30-34
l=1	1.86E+05	1.51E+06	3.80E+05	3.67E+05	2.86E+05	4.05E+05	1.40E+05	1.35E+05	6.37E+05	2.95E+05	4.92E+05	7.89E+05
l=2	0	0	0	0	5.00E+03	3.43E+04	1.99E+04	2.26E+04	1.33E+05	2.79E+04	4.83E+04	6.68E+04
l=3	0	0	0	0	2.83E+02	1.94E+03	7.25E+03	7.97E+03	3.25E+04	4.10E+03	4.85E+03	9.12E+03

$i =$	35-39	40-44	45-49	50-54	55-59	60-64	65-69	70-74	75-79	80-84	85
	8.15E+05	7.90E+05	8.25E+05	7.89E+05	6.82E+05	5.67E+05	4.19E+05	3.12E+05	2.50E+05	1.94E+05	1.90E+05
	5.67E+04	4.20E+04	4.39E+04	4.20E+04	3.63E+04	3.02E+04	2.23E+04	1.66E+04	1.33E+04	1.03E+04	1.01E+04
	3.13E+03	4.01E+03	4.18E+03	4.00E+03	3.46E+03	2.88E+03	2.12E+03	1.58E+03	1.27E+03	9.83E+02	9.65E+02

male:

$i =$	0	1-8	9-10	11-12	13-14	15-17	18	19	20-24	25-26	27-29	30-34
l=1	1.93E+05	1.58E+06	3.97E+05	3.85E+05	3.75E+05	5.37E+05	1.72E+05	1.48E+05	6.87E+05	3.07E+05	4.67E+05	7.73E+05
l=2	0	0	0	0	4.10E+03	2.27E+04	1.64E+04	2.99E+04	1.78E+05	4.83E+04	6.39E+04	6.44E+04
l=3	0	0	0	0	1.10E+03	6.09E+03	2.82E+03	1.10E+04	4.44E+04	9.63E+03	2.09E+04	2.53E+04

$i =$	35-39	40-44	45-49	50-54	55-59	60-64	65-69	70-74	75-79	80-84	85
	7.67E+05	7.69E+05	7.95E+05	7.52E+05	6.28E+05	5.14E+05	3.66E+05	2.58E+05	1.89E+05	1.26E+05	9.17E+04
	6.85E+04	5.20E+04	5.37E+04	5.08E+04	4.24E+04	3.47E+04	2.48E+04	1.74E+04	1.28E+04	8.54E+03	6.20E+03
	2.04E+04	6.00E+03	6.20E+03	5.87E+03	4.90E+03	4.01E+03	2.86E+03	2.01E+03	1.48E+03	9.86E+02	7.16E+02

Vaccinated with 1 dose: V1

female:

i =	13-14	15-17	18	19	20-24	25-26	else
l=1	2.42E+04	3.42E+04	4.62E+03	4.47E+03	2.11E+04	9.76E+03	0
l=2	4.23E+02	2.90E+03	6.57E+02	7.48E+02	4.41E+03	9.23E+02	0
l=3	2.39E+01	1.64E+02	2.40E+02	2.63E+02	1.07E+03	1.36E+02	0

male:

i =	19	20-24	25-26	else
l=1	6.05E+01	6.34E+02	2.83E+02	0
l=2	1.23E+01	1.64E+02	4.46E+01	0
l=3	4.51E+00	4.10E+01	8.89E+00	0

Vaccinated with 2 doses: V2

female:

i =	13-14	15-17	18	19	20-24	25-26	else
l=1	4.88E+04	6.89E+04	9.09E+03	8.79E+03	4.14E+04	1.92E+04	0
l=2	8.53E+02	5.84E+03	1.29E+03	1.47E+03	8.68E+03	1.82E+03	0
l=3	4.82E+01	3.30E+02	4.71E+02	5.18E+02	2.11E+03	2.67E+02	0

male:

i =	19	20-24	25-26	else
l=1	1.36E+02	1.20E+03	5.35E+02	0
l=2	2.76E+01	3.10E+02	8.43E+01	0
l=3	1.02E+01	7.74E+01	1.68E+01	0

Vaccinated with waned immunity: VS=0

Infected: Y

female:

i =	0	1-8	9-10	11-12	13-14	15-17	18	19	20-24	25-26	27-29	30-34
l=1	0	0	0	0	0	7.04E+03	2.16E+03	2.09E+03	9.83E+03	4.55E+03	6.99E+03	1.12E+04
l=2	0	0	0	0	0	5.97E+02	3.07E+02	3.49E+02	2.06E+03	4.31E+02	6.86E+02	9.48E+02
l=3	0	0	0	0	0	3.37E+01	1.12E+02	1.23E+02	5.01E+02	6.33E+01	6.89E+01	1.29E+02

i =	35-39	40-44	45-49	50-54	55-59	60-64	65-69	70-74	75-79	80-84	85
l=1	1.16E+04	1.12E+04	1.17E+04	1.12E+04	9.68E+03	8.06E+03	5.95E+03	4.44E+03	3.55E+03	2.75E+03	2.70E+03
l=2	8.05E+02	5.97E+02	6.23E+02	5.96E+02	5.15E+02	4.28E+02	3.16E+02	2.36E+02	1.89E+02	1.46E+02	1.44E+02
l=3	4.45E+01	5.69E+01	5.94E+01	5.68E+01	4.91E+01	4.08E+01	3.02E+01	2.25E+01	1.80E+01	1.40E+01	1.37E+01

male:

i =	0	1-8	9-10	11-12	13-14	15-17	18	19	20-24	25-26	27-29	30-34
l=1	0	0	0	0	0	1.26E+04	4.06E+03	3.48E+03	1.62E+04	7.24E+03	1.10E+04	1.82E+04
l=2	0	0	0	0	0	5.35E+02	3.85E+02	7.06E+02	4.20E+03	1.14E+03	1.50E+03	1.52E+03
l=3	0	0	0	0	0	1.43E+02	6.63E+01	2.60E+02	1.05E+03	2.27E+02	4.91E+02	5.95E+02

i =	35-39	40-44	45-49	50-54	55-59	60-64	65-69	70-74	75-79	80-84	85
l=1	1.81E+04	1.81E+04	1.87E+04	1.77E+04	1.48E+04	1.21E+04	8.62E+03	6.07E+03	4.46E+03	2.97E+03	2.16E+03
l=2	1.61E+03	1.22E+03	1.26E+03	1.20E+03	9.99E+02	8.18E+02	5.83E+02	4.10E+02	3.01E+02	2.01E+02	1.46E+02
l=3	4.79E+02	1.41E+02	1.46E+02	1.38E+02	1.15E+02	9.44E+01	6.73E+01	4.74E+01	3.48E+01	2.32E+01	1.69E+01

Recovered without sero-conversion: ZS

female:

i =	0	1-8	9-10	11-12	13-14	15-17	18	19	20-24	25-26	27-29	30-34
l=1	0	0	0	0	0	2.37E+03	7.25E+02	7.01E+02	3.30E+03	1.53E+03	2.35E+03	3.77E+03
l=2	0	0	0	0	0	2.01E+02	1.03E+02	1.17E+02	6.92E+02	1.45E+02	2.31E+02	3.19E+02
l=3	0	0	0	0	0	1.13E+01	3.76E+01	4.13E+01	1.68E+02	2.13E+01	2.32E+01	4.35E+01

i =	35-39	40-44	45-49	50-54	55-59	60-64	65-69	70-74	75-79	80-84	85
l=1	3.89E+03	3.77E+03	3.94E+03	3.77E+03	3.25E+03	2.71E+03	2.00E+03	1.49E+03	1.19E+03	9.26E+02	9.08E+02
l=2	2.71E+02	2.01E+02	2.09E+02	2.00E+02	1.73E+02	1.44E+02	1.06E+02	7.93E+01	6.36E+01	4.92E+01	4.83E+01
l=3	1.49E+01	1.91E+01	2.00E+01	1.91E+01	1.65E+01	1.37E+01	1.01E+01	7.56E+00	6.06E+00	4.69E+00	4.60E+00

male:

i =	0	1-8	9-10	11-12	13-14	15-17	18	19	20-24	25-26	27-29	30-34
l=1	0	0	0	0	0	1.01E+04	3.25E+03	2.78E+03	1.30E+04	5.79E+03	8.80E+03	1.46E+04
l=2	0	0	0	0	0	4.28E+02	3.08E+02	5.65E+02	3.36E+03	9.13E+02	1.20E+03	1.21E+03
l=3	0	0	0	0	0	1.15E+02	5.30E+01	2.08E+02	8.38E+02	1.82E+02	3.93E+02	4.76E+02

i =	35-39	40-44	45-49	50-54	55-59	60-64	65-69	70-74	75-79	80-84	85
l=1	1.44E+04	1.45E+04	1.50E+04	1.42E+04	1.18E+04	9.68E+03	6.90E+03	4.86E+03	3.57E+03	2.38E+03	1.73E+03
l=2	1.29E+03	9.79E+02	1.01E+03	9.58E+02	8.00E+02	6.54E+02	4.66E+02	3.28E+02	2.41E+02	1.61E+02	1.17E+02
l=3	3.84E+02	1.13E+02	1.17E+02	1.11E+02	9.23E+01	7.56E+01	5.38E+01	3.79E+01	2.78E+01	1.86E+01	1.35E+01

Recovered with sero-conversion: Z

female:

i =	0	1-8	9-10	11-12	13-14	15-17	18	19	20-24	25-26	27-29	30-34
l=1	0	0	0	0	0	3.48E+03	1.06E+03	1.03E+03	4.85E+03	2.25E+03	3.45E+03	5.53E+03
l=2	0	0	0	0	0	2.95E+02	1.52E+02	1.72E+02	1.02E+03	2.13E+02	3.39E+02	4.68E+02
l=3	0	0	0	0	0	1.67E+01	5.52E+01	6.07E+01	2.47E+02	3.12E+01	3.40E+01	6.39E+01

i =	35-39	40-44	45-49	50-54	55-59	60-64	65-69	70-74	75-79	80-84	85
l=1	5.72E+03	5.54E+03	5.79E+03	5.53E+03	4.78E+03	3.98E+03	2.94E+03	2.19E+03	1.76E+03	1.36E+03	1.33E+03
l=2	3.98E+02	2.95E+02	3.08E+02	2.94E+02	2.54E+02	2.12E+02	1.56E+02	1.16E+02	9.34E+01	7.23E+01	7.10E+01
l=3	2.20E+01	2.81E+01	2.93E+01	2.81E+01	2.42E+01	2.02E+01	1.49E+01	1.11E+01	8.90E+00	6.90E+00	6.76E+00

male:

$i =$	0	1-8	9-10	11-12	13-14	15-17	18	19	20-24	25-26	27-29	30-34
l=1	0	0	0	0	0	3.78E+02	1.21E+02	1.04E+02	4.84E+02	2.16E+02	3.29E+02	5.44E+02
l=2	0	0	0	0	0	1.60E+01	1.15E+01	2.11E+01	1.25E+02	3.41E+01	4.49E+01	4.53E+01
l=3	0	0	0	0	0	4.29E+00	1.98E+00	7.76E+00	3.13E+01	6.79E+00	1.47E+01	1.78E+01
$i =$	35-39	40-44	45-49	50-54	55-59	60-64	65-69	70-74	75-79	80-84	85	
l=1	5.40E+02	5.41E+02	5.59E+02	5.29E+02	4.42E+02	3.62E+02	2.58E+02	1.81E+02	1.33E+02	8.88E+01	6.45E+01	
l=2	4.82E+01	3.66E+01	3.78E+01	3.58E+01	2.99E+01	2.44E+01	1.74E+01	1.23E+01	9.01E+00	6.01E+00	4.36E+00	
l=3	1.43E+01	4.22E+00	4.36E+00	4.13E+00	3.45E+00	2.82E+00	2.01E+00	1.42E+00	1.04E+00	6.93E-01	5.03E-01	

Infected vaccinated with 1 dose: W1

female:

$i =$	15-17	18	19	20-24	25-26	else
l=1	2.69E+03	3.63E+02	3.51E+02	1.65E+03	7.67E+02	0
l=2	2.28E+02	5.17E+01	5.88E+01	3.47E+02	7.26E+01	0
l=3	1.29E+01	1.88E+01	2.07E+01	8.44E+01	1.07E+01	0

male:

$i =$	19	20-24	25-26	else
l=1	4.75E+00	4.98E+01	2.23E+01	0
l=2	9.65E-01	1.29E+01	3.51E+00	0
l=3	3.55E-01	3.22E+00	6.99E-01	0

Infected vaccinated with 2 dose: W2

female:

$i =$	15-17	18	19	20-24	25-26	else
l=1	5.41E+03	7.14E+02	6.91E+02	3.25E+03	1.51E+03	0
l=2	4.59E+02	1.02E+02	1.16E+02	6.82E+02	1.43E+02	0
l=3	2.60E+01	3.71E+01	4.07E+01	1.66E+02	2.10E+01	0

male:

$i =$	19	20-24	25-26	else
l=1	1.07E+01	9.41E+01	4.20E+01	0
l=2	2.17E+00	2.44E+01	6.63E+00	0
l=3	7.98E-01	6.08E+00	1.32E+00	0

Infected vaccinated with waned immunity: WS=0

Recovered vaccinated without sero-convertion : QS

female:

$i =$	15-17	18	19	20-24	25-26	else
l=1	2.51E+03	3.33E+02	3.23E+02	1.52E+03	7.04E+02	0
l=2	2.12E+02	4.74E+01	5.40E+01	3.18E+02	6.66E+01	0
l=3	1.20E+01	1.73E+01	1.90E+01	7.75E+01	9.78E+00	0

male:

$i =$	19	20-24	25-26	else
l=1	1.14E+01	1.06E+02	4.73E+01	0
l=2	2.31E+00	2.75E+01	7.47E+00	0
l=3	8.49E-01	6.85E+00	1.49E+00	0

Recovered vaccinated with sero-convertion : Q

female:

$i =$	15-17	18	19	20-24	25-26	else
l=1	3.68E+03	4.90E+02	4.74E+02	2.23E+03	1.03E+03	0
l=2	3.12E+02	6.97E+01	7.93E+01	4.68E+02	9.79E+01	0
l=3	1.77E+01	2.54E+01	2.79E+01	1.14E+02	1.44E+01	0

male:

$i =$	19	20-24	25-26	else
l=1	4.25E-01	3.96E+00	1.77E+00	0
l=2	8.62E-02	1.03E+00	2.79E-01	0
l=3	3.17E-02	2.56E-01	5.55E-02	0

Total number of persons: N

female:

$i =$	0	1-8	9-10	11-12	13-14	15-17	18	19	20-24	25-26	27-29	30-34
l=1	1.86e+05	1.51e+06	3.80e+05	3.67e+05	3.57e+05	5.03e+05	1.54e+05	1.49e+05	7.02e+05	3.25e+05	4.99e+05	8.01e+05
l=2	0	0	0	0	6.23e+03	4.26e+04	2.19e+04	2.49e+04	1.47e+05	3.08e+04	4.90e+04	6.77e+04
l=3	0	0	0	0	3.52e+02	2.41e+03	7.99e+03	8.78e+03	3.58e+04	4.52e+03	4.92e+03	9.25e+03

$i =$	35-39	40-44	45-49	50-54	55-59	60-64	65-69	70-74	75-79	80-84	85
	8.27e+05	8.01e+05	8.37e+05	8.00e+05	6.91e+05	5.75e+05	4.25e+05	3.17e+05	2.54e+05	1.97e+05	1.93e+05
	5.75e+04	4.26e+04	4.45e+04	4.26e+04	3.68e+04	3.06e+04	2.26e+04	1.68e+04	1.35e+04	1.05e+04	1.03e+04
	3.18e+03	4.06e+03	4.24e+03	4.06e+03	3.51e+03	2.92e+03	2.16e+03	1.61e+03	1.29e+03	9.97e+02	9.78e+02

male:

$i =$	0	1-8	9-10	11-12	13-14	15-17	18	19	20-24	25-26	27-29	30-34
l=1	1.93e+05	1.58e+06	3.97e+05	3.85e+05	3.75e+05	5.50e+05	1.76e+05	1.51e+05	7.04e+05	3.15e+05	4.78e+05	7.91e+05
l=2	0	0	0	0	4.10e+03	2.32e+04	1.67e+04	3.07e+04	1.82e+05	4.96e+04	6.54e+04	6.60e+04
l=3	0	0	0	0	1.10e+03	6.24e+03	2.88e+03	1.13e+04	4.55e+04	9.88e+03	2.13e+04	2.59e+04

$i =$	35-39	40-44	45-49	50-54	55-59	60-64	65-69	70-74	75-79	80-84	85
	7.85e+05	7.87e+05	8.14e+05	7.70e+05	6.43e+05	5.26e+05	3.75e+05	2.64e+05	1.94e+05	1.29e+05	9.39e+04
	7.02e+04	5.32e+04	5.50e+04	5.20e+04	4.34e+04	3.56e+04	2.53e+04	1.78e+04	1.31e+04	8.74e+03	6.35e+03
	2.08e+04	6.14e+03	6.35e+03	6.01e+03	5.02e+03	4.11e+03	2.93e+03	2.06e+03	1.51e+03	1.01e+03	7.33e+02

Detected local oropharyngeal cancer: DOPCl

female:

$i =$	0	1-8	9-10	11-12	13-14	15-17	18	19	20-24	25-26	27-29	30-34
l=1	0	0	0	0	0	0	0	0	0	9.02E-05	9.03E-05	9.12E-05
l=2	0	0	0	0	0	0	0	0	0	8.53E-06	8.85E-06	7.72E-06
l=3	0	0	0	0	0	0	0	0	0	1.25E-06	8.89E-07	1.05E-06
$i =$	35-39	40-44	45-49	50-54	55-59	60-64	65-69	70-74	75-79	80-84	85	
	1.86E-03	1.42E-03	4.25E-03	5.48E-03	5.76E-03	1.06E-02	1.56E-02	1.73E-02	2.11E-02	2.03E-02	1.37E-02	
	1.30E-04	7.54E-05	2.26E-04	2.91E-04	3.07E-04	5.63E-04	8.29E-04	9.20E-04	1.12E-03	1.08E-03	7.29E-04	
	7.16E-06	7.19E-06	2.16E-05	2.78E-05	2.92E-05	5.37E-05	7.90E-05	8.77E-05	1.07E-04	1.03E-04	6.95E-05	

male:

$i =$	0	1-8	9-10	11-12	13-14	15-17	18	19	20-24	25-26	27-29	30-34
l=1	0	0	0	0	0	0	0	0	1.51E-04	2.52E-04	2.54E-04	7.17E-03
l=2	0	0	0	0	0	0	0	0	3.91E-05	3.98E-05	3.47E-05	5.98E-04
l=3	0	0	0	0	0	0	0	0	9.77E-06	7.92E-06	1.13E-05	2.35E-04
$i =$	35-39	40-44	45-49	50-54	55-59	60-64	65-69	70-74	75-79	80-84	85	
	8.96E-04	1.86E-03	6.04E-03	1.12E-02	2.18E-02	2.74E-02	3.70E-02	3.70E-02	3.29E-02	3.31E-02	2.99E-02	
	8.01E-05	1.26E-04	4.09E-04	7.54E-04	1.47E-03	1.85E-03	2.50E-03	2.50E-03	2.22E-03	2.24E-03	2.02E-03	
	2.38E-05	1.45E-05	4.72E-05	8.71E-05	1.70E-04	2.14E-04	2.89E-04	2.89E-04	2.57E-04	2.58E-04	2.34E-04	

Detected regional oropharyngeal cancer: DOPCr

female:

$i =$	0	1-8	9-10	11-12	13-14	15-17	18	19	20-24	25-26	27-29	30-34
l=1	0	0	0	0	0	0	0	0	0	1.80E-04	1.81E-04	2.74E-04
l=2	0	0	0	0	0	0	0	0	0	1.71E-05	1.77E-05	2.32E-05
l=3	0	0	0	0	0	0	0	0	0	2.51E-06	1.78E-06	3.16E-06
$i =$	35-39	40-44	45-49	50-54	55-59	60-64	65-69	70-74	75-79	80-84	85	
	1.12E-03	2.55E-03	5.76E-03	9.92E-03	1.38E-02	1.64E-02	1.92E-02	2.13E-02	1.54E-02	1.27E-02	6.90E-03	
	7.78E-05	1.36E-04	3.07E-04	5.28E-04	7.34E-04	8.74E-04	1.02E-03	1.13E-03	8.19E-04	6.73E-04	3.67E-04	
	4.29E-06	1.29E-05	2.92E-05	5.03E-05	6.99E-05	8.34E-05	9.73E-05	1.08E-04	7.81E-05	6.42E-05	3.50E-05	

male:

$i =$	0	1-8	9-10	11-12	13-14	15-17	18	19	20-24	25-26	27-29	30-34
l=1	0	0	0	0	0	0	0	0	0	2.52E-04	2.54E-04	5.38E-03
l=2	0	0	0	0	0	0	0	0	0	3.98E-05	3.47E-05	4.48E-04
l=3	0	0	0	0	0	0	0	0	0	7.92E-06	1.13E-05	1.76E-04
$i =$	35-39	40-44	45-49	50-54	55-59	60-64	65-69	70-74	75-79	80-84	85	
	2.06E-03	8.56E-03	2.02E-02	5.37E-02	8.40E-02	1.07E-01	1.03E-01	8.63E-02	7.15E-02	3.98E-02	2.82E-02	
	1.84E-04	5.78E-04	1.36E-03	3.63E-03	5.68E-03	7.21E-03	6.95E-03	5.83E-03	4.83E-03	2.69E-03	1.90E-03	
	5.47E-05	6.68E-05	1.57E-04	4.19E-04	6.55E-04	8.32E-04	8.02E-04	6.73E-04	5.58E-04	3.11E-04	2.20E-04	

Detected distant oropharyngeal cancer: DOPCd

female:

$i =$	0	1-8	9-10	11-12	13-14	15-17	18	19	20-24	25-26	27-29	30-34
l=1	0	0	0	0	0	0	0	0	0	0	0	0
l=2	0	0	0	0	0	0	0	0	0	0	0	0
l=3	0	0	0	0	0	0	0	0	0	0	0	0
$i =$	35-39	40-44	45-49	50-54	55-59	60-64	65-69	70-74	75-79	80-84	85	
	0	0	1.13E-03	1.51E-03	2.17E-03	2.46E-03	2.27E-03	2.27E-03	1.42E-03	2.27E-03	1.42E-03	
	0	0	6.03E-05	8.04E-05	1.16E-04	1.31E-04	1.21E-04	1.21E-04	7.54E-05	1.21E-04	7.54E-05	
	0	0	5.75E-06	7.67E-06	1.10E-05	1.25E-05	1.15E-05	1.15E-05	7.19E-06	1.15E-05	7.19E-06	

male:

$i =$	0	1-8	9-10	11-12	13-14	15-17	18	19	20-24	25-26	27-29	30-34
l=1	0	0	0	0	0	0	0	0	0	8.41E-05	8.46E-05	1.79E-04
l=2	0	0	0	0	0	0	0	0	0	1.33E-05	1.16E-05	1.49E-05
l=3	0	0	0	0	0	0	0	0	0	2.64E-06	3.78E-06	5.86E-06
$i =$	35-39	40-44	45-49	50-54	55-59	60-64	65-69	70-74	75-79	80-84	85	
	8.96E-05	1.39E-03	3.91E-03	7.63E-03	1.39E-02	1.69E-02	1.63E-02	1.07E-02	1.02E-02	6.60E-03	2.70E-03	
	8.01E-06	9.43E-05	2.64E-04	5.15E-04	9.36E-04	1.14E-03	1.10E-03	7.23E-04	6.91E-04	4.46E-04	1.82E-04	
	2.38E-06	1.09E-05	3.05E-05	5.95E-05	1.08E-04	1.32E-04	1.27E-04	8.34E-05	7.98E-05	5.15E-05	2.10E-05	

Oropharyngeal cancer survivals for both male and female: SOPC

$i =$	0	1-8	9-10	11-12	13-14	15-17	18	19	20-24	25-26	27-29	30-34
l=1	1	1	1	1	1	1	1	1	8.69E-01	9.09E-01	9.09E-01	9.46E-01
l=2	1	1	1	1	1	1	1	1	8.69E-01	9.09E-01	9.09E-01	9.46E-01
l=3	1	1	1	1	1	1	1	1	8.69E-01	9.09E-01	9.09E-01	9.46E-01
$i =$	35-39	40-44	45-49	50-54	55-59	60-64	65-69	70-74	75-79	80-84	85	
	9.32E-01	9.37E-01	9.33E-01	8.91E-01	9.00E-01	8.74E-01	8.89E-01	8.57E-01	8.40E-01	8.35E-01	8.04E-01	
	9.32E-01	9.37E-01	9.33E-01	8.91E-01	9.00E-01	8.74E-01	8.89E-01	8.57E-01	8.40E-01	8.35E-01	8.04E-01	
	9.32E-01	9.37E-01	9.33E-01	8.91E-01	9.00E-01	8.74E-01	8.89E-01	8.57E-01	8.40E-01	8.35E-01	8.04E-01	
