# Supplementary figures and images for: An economic and disease transmission model of human papillomavirus and oropharyngeal cancer in Texas
Source: Sci Rep. 2021 Jan 19;11:1802. doi: 10.1038/s41598-021-81375-5 (PMC7815750; doi:10.1038/s41598-021-81375-5)

Figure S1: Simplified Schematic Presentation of Models (Part 1)

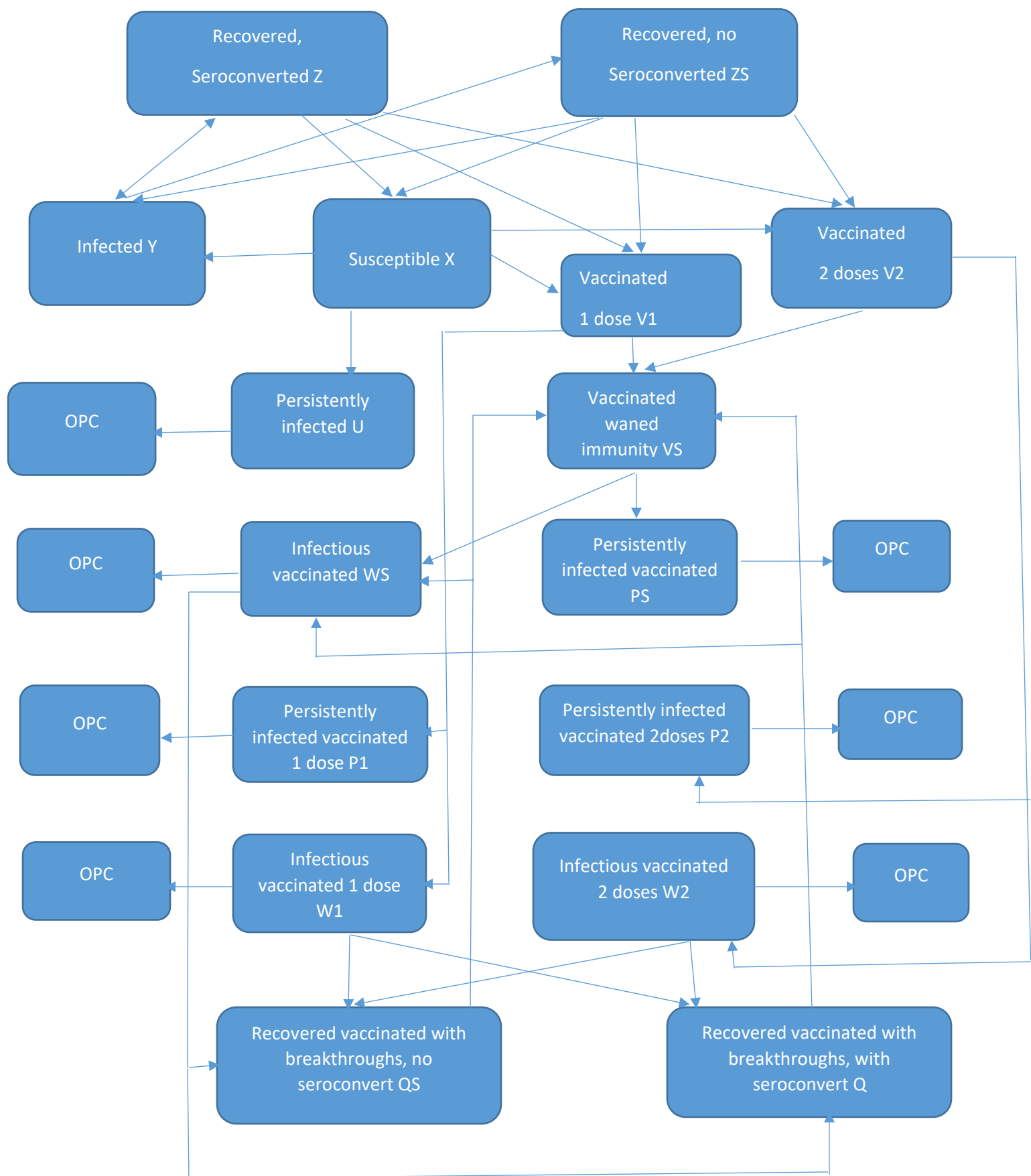

Supplement: Supplementary file 5 — Supplementary Figure S1. [file 41598_2021_81375_MOESM5_ESM.pdf]

Figure S2:Simplified Schematic Presentation of models (Part 2)

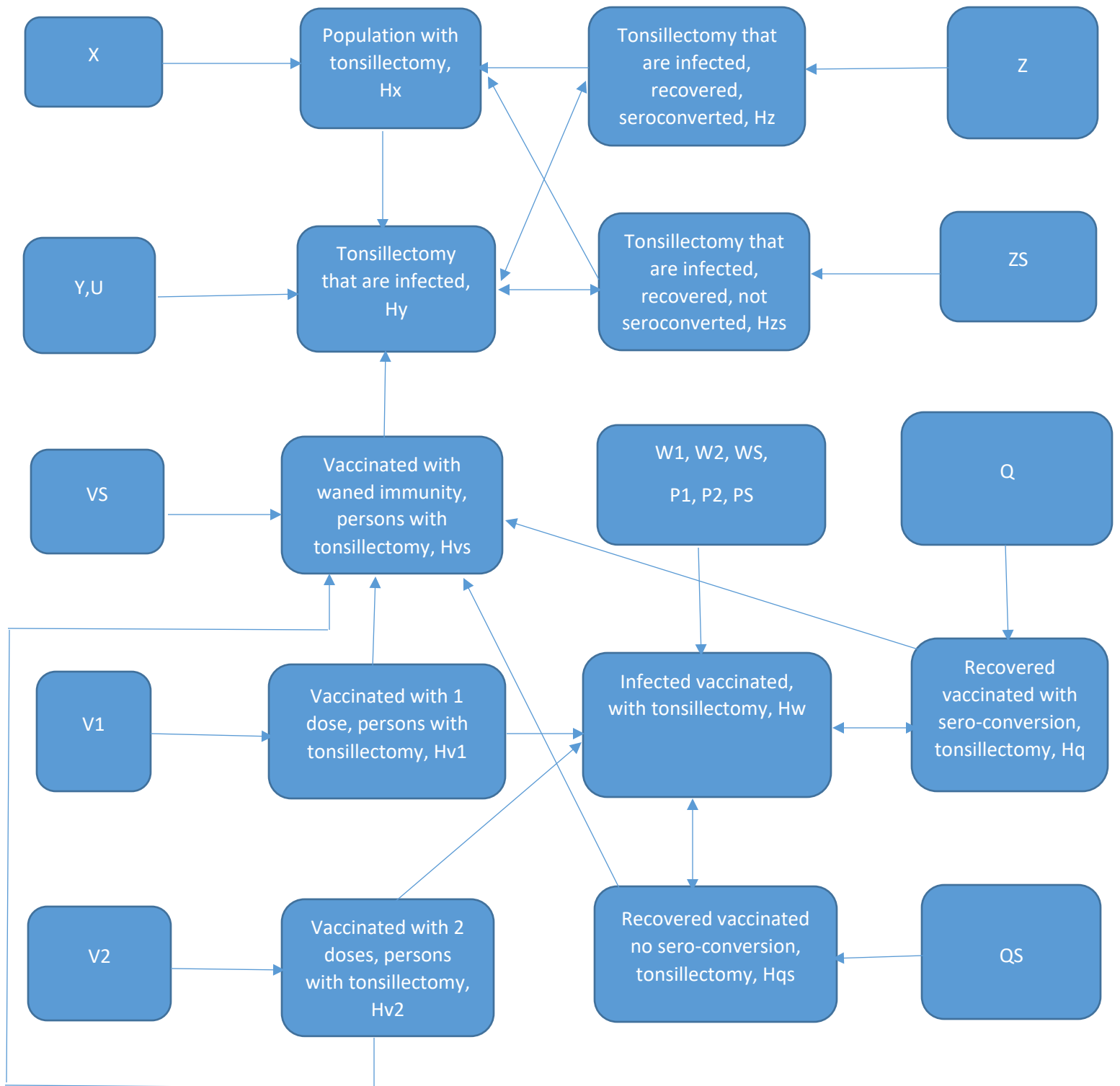

Supplement: Supplementary file 6 — Supplementary Figure S2. [file 41598_2021_81375_MOESM6_ESM.pdf]

Figure S3:Simplified Schematic Presentation of models (Part 3)

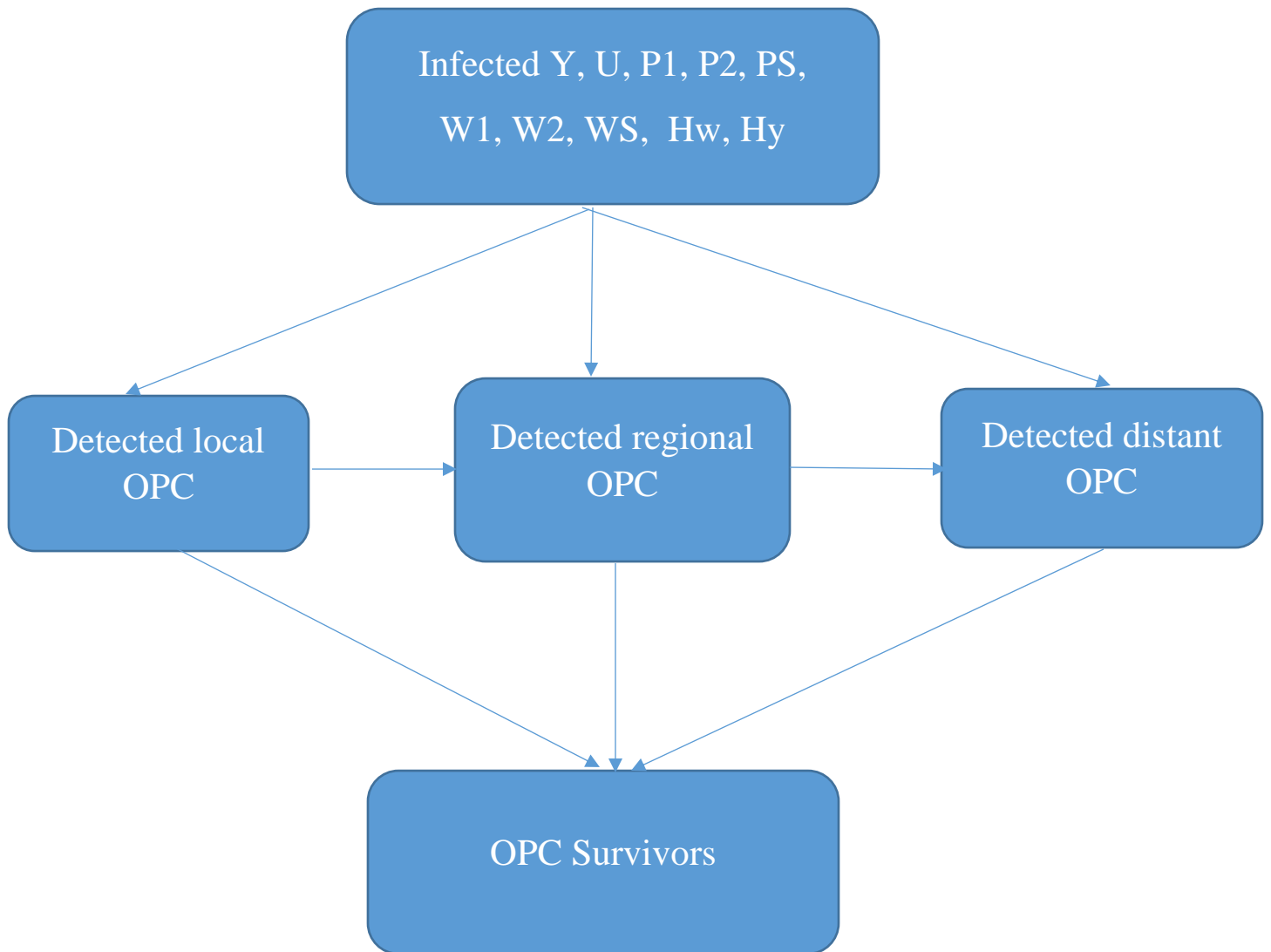

Supplement: Supplementary file 7 — Supplementary Figure S3. [file 41598_2021_81375_MOESM7_ESM.pdf]

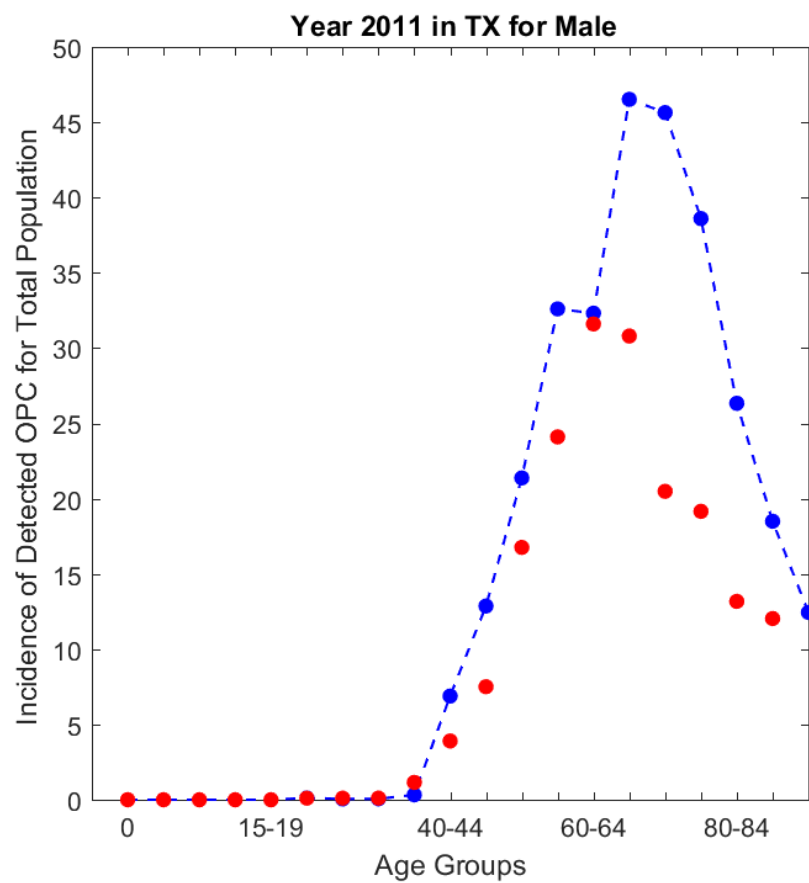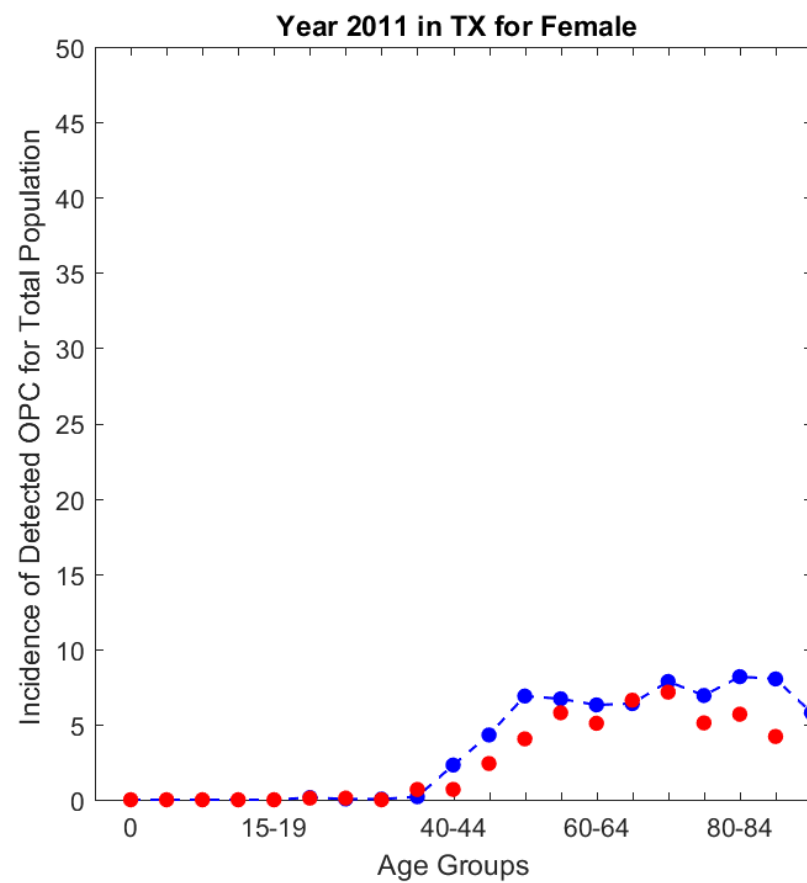

Supplement: Supplementary file 8 — Supplementary Figure S4. [file 41598_2021_81375_MOESM8_ESM.pdf]

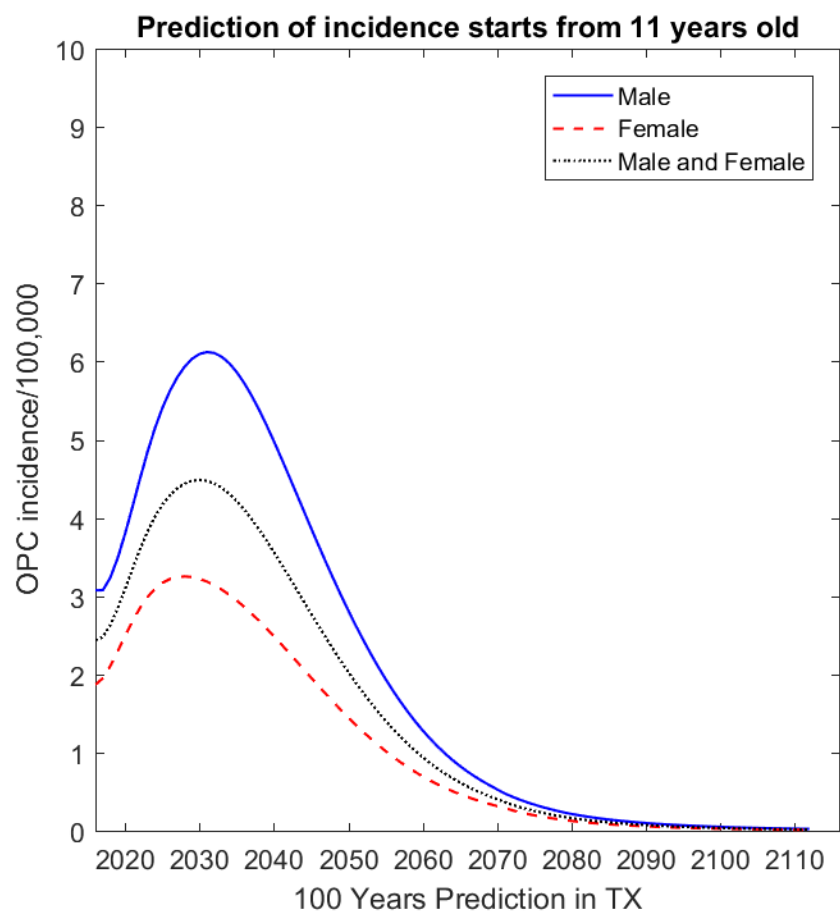

Supplement: Supplementary file 9 — Supplementary Figure S5. [file 41598_2021_81375_MOESM9_ESM.pdf]

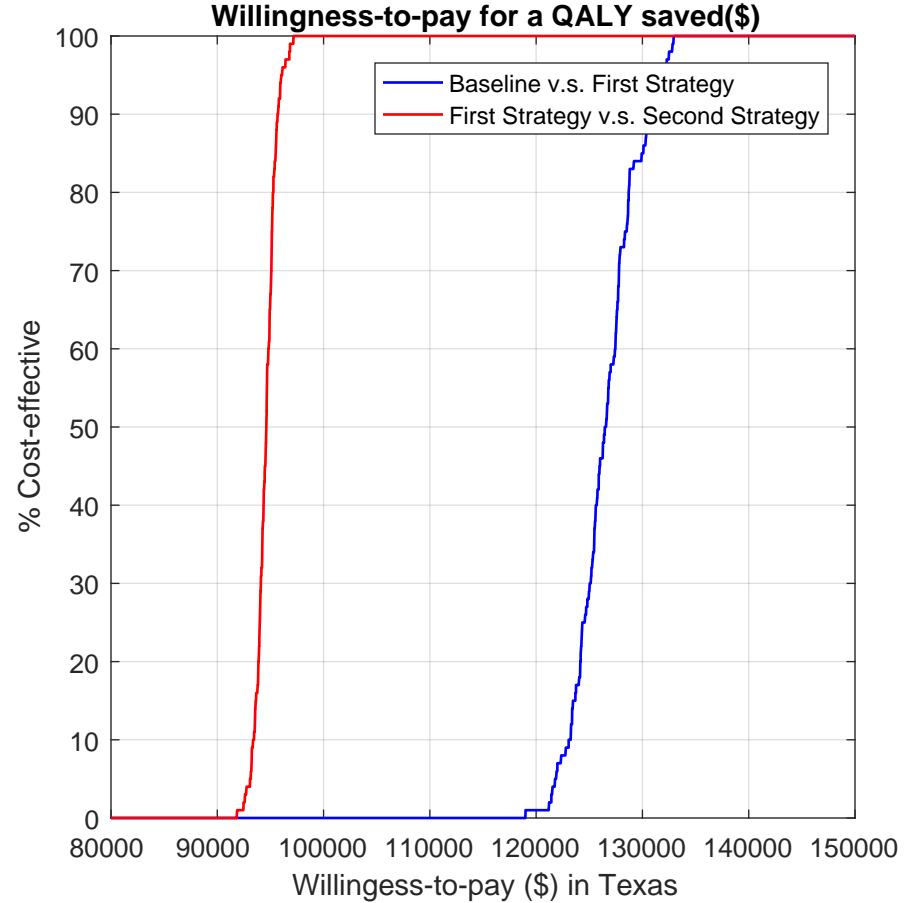

Supplement: Supplementary file 10 — Supplementary Figure S6. [file 41598_2021_81375_MOESM10_ESM.pdf]

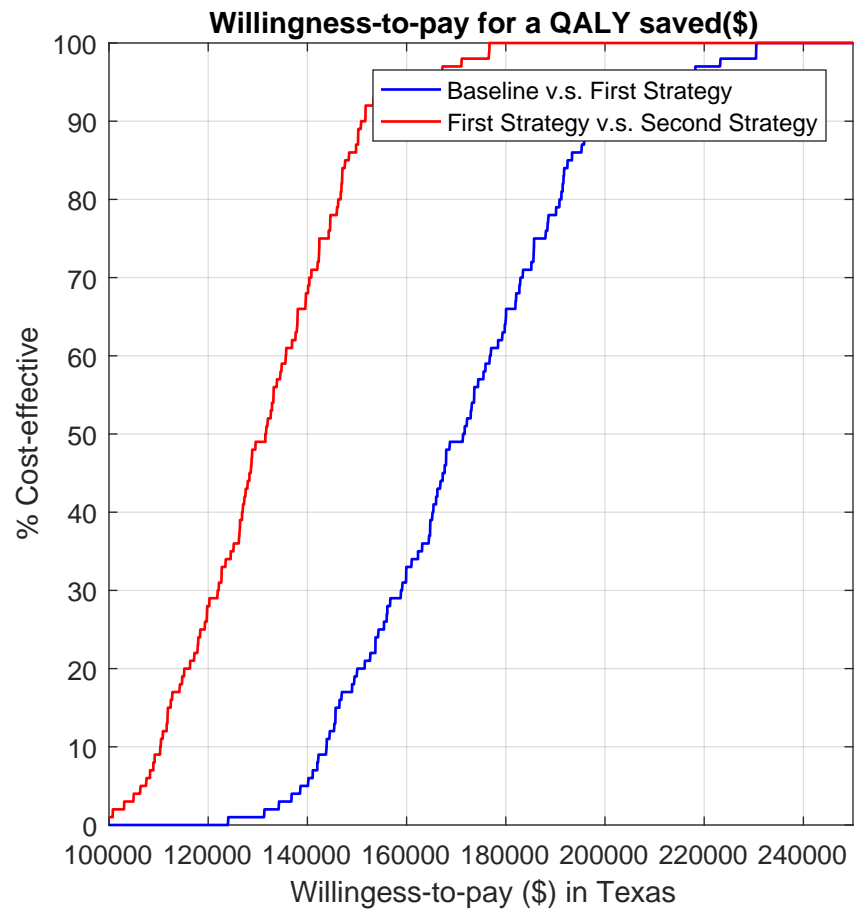

Supplement: Supplementary file 11 — Supplementary Figure S7. [file 41598_2021_81375_MOESM11_ESM.pdf]
